# Endothelial dysfunction in Fabry disease: retinal biomarkers link cardiac GLA gene variants with chronic inflammation

**DOI:** 10.1038/s41525-025-00540-1

**Published:** 2026-01-16

**Authors:** Timon Wallraven, Claudia Regenbogen, Roman Günthner, Andrea Ribeiro, Javier Carbajo-Lozoya, Nora Hannane, Michael Wunderle, Abdelrahman Assaf, Maciej Lech, Henner Hanssen, Lukas Streese, Derralynn Hughes, Bernhard Haller, Konstantin Kotliar, Uwe Heemann, Christoph Schmaderer

**Affiliations:** 1 TUM School of Medicine and Health, Department of Nephrology, TUM University Hospital, Technical University of Munich, Munich, Germany; 2 Medizinische Klinik und Poliklinik IV, LMU University Hospital Munich, Munich, Germany; 3 Department of Sport, Exercise and Health, Preventive Sports Medicine and Systems Physiology, University of Basel, Basel, Switzerland; 4 Department of Nephrology, Medical Faculty, University Hospital Düsseldorf, Heinrich-Heine-University Düsseldorf, Düsseldorf, Germany; 5 Lysosomal Storage Disorders Unit, The Royal Free London NHS Foundation Trust, University College London, London, United Kingdom; 6 TUM School of Medicine and Health, Institute of AI and Informatics in Medicine, TUM University Hospital, Technical University of Munich, Munich, Germany; 7 Aachen University of Applied Sciences, Jülich, Germany; 8 German Centre for Infection Research (DZIF), Partner Site Munich, Munich, Germany

**Keywords:** Genetics research, Biomarkers, Disease genetics

## Abstract

Fabry disease (FD) is a rare X-linked lysosomal storage disorder caused by variants in the alpha-galactosidase A gene (GLA). Cardiac complications are a major cause of mortality, but the large number of variants complicate early identification of at-risk patients. In this study, we assessed the microcirculation using Retinal Vessel Analysis (RVA) in 63 FD patients age- and gender-matched to 60 healthy controls, analyzing associations between RVA parameters, cardiac involvement, and GLA variants. FD patients showed reduced venular flicker-induced dilation, narrower retinal arterioles, and a lower arteriolar-to-venular ratio. Impaired retinal microcirculation was associated with cardiac involvement, and patients with cardiac-associated GLA variants exhibited narrower retinal arterioles. Markers of inflammation and endothelial dysfunction (ED) were significantly higher in FD patients. Higher inflammatory levels correlated with altered retinal microcirculation in patients carrying cardiac-associated GLA variants. RVA detects microvascular ED in FD patients and may serve as a non-invasive biomarker for cardiovascular risk stratification. Registration**:** https://clinicaltrials.gov/study/NCT06758648; Unique identifier: NCT06758648.

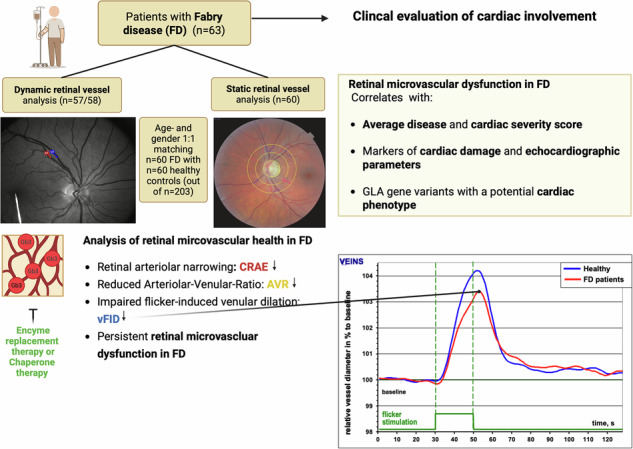

## Introduction

Fabry disease (FD, OMIM-301500) is a rare, X-linked lysosomal storage disorder with a reduced or completely absent activity of the enzyme α-galactosidase A encoded by the GLA-gene. This malfunction leads to a progressive accumulation of glycosphingolipids, such as globotriaosylceramide (Gb3), in the endothelium of different organs. Primary cellular dysfunction and secondary inflammation result in reduced organ perfusion and fibrosis, which causes progressive, multisystemic organ damage^1–3^. In adult FD patients, cardiovascular manifestations, including left ventricular hypertrophy (LVH), and cerebrovascular complications, including stroke, contribute to a significantly lower life expectancy^4–6^. Heterozygous females with α-galactosidase A deficiency may also develop Fabry-associated complications, although usually with a slower progression^7^. Diagnostic delay of FD can reduce the efficacy of the treatment, and cardiovascular complications may significantly benefit from early treatment^5,8,9^.

Although the exact pathophysiological mechanisms underlying endothelial dysfunction (ED) in FD remain unclear, Gb3 has been shown to induce the degradation of endothelial K_Ca_ 3.1 channels, which play a crucial role in endothelium-dependent hyperpolarization. Additionally, secondary chronic inflammation may contribute to the degradation of the endothelial glycocalyx, further exacerbating ED in FD patients^10,11^. Consequently, various markers of endothelial activation and inflammation, including vascular endothelial growth factor (VEGF), intercellular adhesion molecule-1 (ICAM-1), and monocyte chemoattractant protein-1 (MCP1), are elevated in patients and markers of inflammation are associated with cardiac disease progression in FD^12–16^. After the administration of enzyme replacement therapy (ERT), complete microvascular clearance of Gb3 in the endothelium and podocytes has been observed^17,18^. However, in advanced FD patients with severe LVH, intracellular clearance of glycosphingolipids in cardiomyocytes appears to be ineffective despite ERT infusion due to fibrosis and inflammation^19^. Furthermore, Gb3 clearance may not prevent altered molecular signaling, further underscoring the importance of early disease detection^17^.

Quantifying ED by non-invasive imaging methods has been proposed by the European Society of Cardiology (ESC)^20^, and studies have shown impaired endothelial function in medium- to large-sized vessels via pulse-wave velocity (PWV) and flow-mediated dilation (FMD)^21,22^. However, cardiovascular complications in FD are mainly a result of coronary microvascular dysfunction, and monitoring the microcirculation with non-invasive tools could potentially provide a better tool for early diagnosis and disease monitoring^18,23,24^. To our knowledge to date, there is no functional data on small vessel health in FD.

Dynamic retinal vessel analysis (DVA) and static retinal vessel analysis (SVA) are two quantitative and non-invasive technologies that follow up on changes in retinal microcirculation as a predictive tool for the progression of systemic cardiovascular disease (CVD). DVA quantifies the reaction to flickering light over time, mainly mediated by neuro-vascular coupling followed by shear-stress-induced nitric oxide (NO) release^25^. This allows direct assessment of the cerebrovascular function, and especially in CVD, DVA and SVA have shown in multiple studies their high potential to monitor endothelial health non-invasively^26,27^. The Atherosclerosis Risk in Communities (ARIC) study proved the excellent predictive value of retinal arteriolar narrowing and venular widening as a long-term predictor of CV events^28^. In a large group of dialysis patients, we were able to show that impaired retinal venular dilation (vFID) is an independent predictor of all-cause mortality^29^.

Early detection of organ damage in FD is crucial, however diagnosis in early stages still presents a considerable challenge. Therefore, the study’s primary aim was to investigate whether the retinal structure and function, measured by DVA and SVA, are altered in FD. The secondary aim was to evaluate how parameters of the retinal microcirculation are associated with clinical disease severity, ERT and/or pharmacological chaperone therapy (PCT), and the cardiac phenotype.

## Results

### Patients’ characteristics and subgroup analysis

Out of 63 FD patients, 60 (95.2%) had high-quality SVA images (68.3% female, mean age 48.6 ± 16.5 years) and were age- and gender-matched with 60 healthy volunteers (68.3% female, 49.7 ± 16.2 years) from a previously recruited HC (n = 204)^30^. The occurrence of CV risk factors was comparable between cohorts after matching. There was a tendency towards more nicotine abuse in FD patients; however, not significantly. The most frequent CV risk factors in FD patients were hypercholesterolemia (53.3%) and obesity (36.7%). Fabry-related complications included patients with left ventricular hypertrophy (LVH, 48.3%), patients with central nervous, vascular disease (CNVD, 25.0%), patients with cardiac arrhythmia (21.7%), and patients with chronic kidney disease (CKD, 6.7%). 41 patients (68.3%) were pre-treated with enzyme replacement therapy (ERT) or pharmacological chaperone therapy (PCT; 4/41; 9.8% of treated patients) with a median therapy duration of 1.1 years (0.0–3.4). The average Fabry disease severity scoring system (DS3) was 8.7 (5.2–11.8), and median LysoGb3 levels were 4.3 ng/ml (0.9–8.3) under current ERT/PCT. Laboratory parameters showed significantly higher leukocytes and lower hemoglobin levels in FD patients (Table 1).

**Table 1:** Characteristics of Fabry disease patients compared to age- and gender-matched healthy controls

Clinical characteristics	FD patients (*n* = 60)	HC (*n* = 60)	*P* value
Age			
y, mean (SD)	48.6 (±16.5)	49.7 (±16.2)	0.72
Sex			
female	41 (68.3%)	41 (68.3%)	-
BMI			
kg/m^2^, mean (IQR)	24.1 (21.6–27.8)	23.0 (21.1–25.8)	0.28
Cardiovascular risk factors			
Obesity	22 (36.7%)	17 (28.3%)	0.44
Hypercholesterolemia	32 (53.3%)	-	-
Arterial hypertension	21 (35.0%)	15 (25.0%)	0.32
Diabetes mellitus	0 (0%)	0 (0%)	-
Nicotine abuse	17 (28.3%)	6 (10.0%)	0.078
RR MAD			
mmHg, Mean (IQR)	98.5 (93.0–107.8)	100.0 (96.0–107.0)	0.47
Fabry-related complications			
LVH	29 (48.3%)	-	-
CNVD	15 (25.0%)	-	-
CKD	4 (6.7%)	-	-
Cardiac arrhythmia	13 (21.7%)	-	-
Fabry disease severity and treatment			
ERT or PCT	41 (68.3%)	-	-
Age at therapy start			
y, Median (IQR)	34.0 (0.0–48.8)	-	-
Therapy duration			
y, Median (IQR)	1.1 (0.0–3.4)	-	-
Average DS3			
Median (IQR)	8.7 (5.2–11.8)	-	-
LysoGb3			
ng/ml, median (IQR)	4.3 (0.9–8.3)	-	-
*Quality DVA*			
Quality score veins			
Mean (IQR)	4.5 (3.4–5.0)	4.0 (3.5–4.5)	0.22
Quality score arteriols			
Mean (IQR)	4.0 (3.4–4.5)	3.5 (3.0–4.5)	0.37
*Laboratory parameters*			
Leukocytes			
G/l, median (IQR)	6.3 (5.2–7.4)	5.5 (4.8–6.5)	0.029*
hsCRP			
mg/dl, median (IQR)	0.1 (0.1–0.2)	0.1 (0.0–0.2)	0.097
Hemoglobin			
g/dl, median (IQR)	13.7 (13.1- 14.6)	14.8 (13.6–15.4)	0.0049**
Creatinine			
mg/dl, median (IQR)	0.9 (0.8–0.9)	0.9 (0.8–1.0)	0.45
Phosphate			
mmol/l, mean (SD)	3.4 (±0.5)	3.6 (±0.5)	0.11

*P* values are shown for statistical tests comparing patients with Fabry disease (FD) (*n* = 60) with an age- and gender matched healthy cohort (HC) (*n* = 60); *t* test was used for normally distributed variables, the *χ*^2^ test for categorical variables, the Wilcoxon rank sum test for variables with a skewed distribution, and Fisher’s exact test for binary variables. BMI body mass index, RR MAD the mean arterial pressure, DM diabetes mellitus I or II, obesity is defined as BMI > 25 kg/m^2^, hypercholesterolemia is defined as cholesterol >200 mg/dl. LVH left ventricular hypertrophy is defined as intraventricular thickness >13 mm, CNVD central nervous vascular disease is defined as transient ischemic attack (TIA) or Stroke, ERT Enzyme replacement therapy, PCT Pharmacological chaperone therapy, DVA dynamic retinal vessel analysis, Leukocytes (*n* = 100), Hemoglobin (*n* = 99), Creatinine and Phosphate (both *n* = 103) and high-sensitivity C-reactive protein (hs-CRP, *n* = 97) were measured in our cohort.

Male FD patients (n = 20, mean age 50.4 ± 10.7 years) showed a more severe clinical phenotype than female FD patients (n = 43, mean age 47.4 ± 18.2 years). Frequency of LVH (37.2% vs. 70.0%, p = 0.029), CNVD (13.9% vs. 55.0%, p = 0.0011), and heart valve disease (HVD, 0% vs. 15.0%, p = 0.028) was significantly higher in male patients. Consequently, the ADS3 was significantly higher (7.7 vs. 11.7, p = 0.0052), and male FD patients showed significantly higher levels of LysoGb3 (4.2 vs. 27.0, p = 0.028). CV risk factors and age were not significantly different between male and female FD patients. Ferritin as well as Creatinine levels were significantly elevated in male FD patients (Supplementary Table 1).

### Impaired retinal microvascular function in Fabry disease

**Fig. 1:**
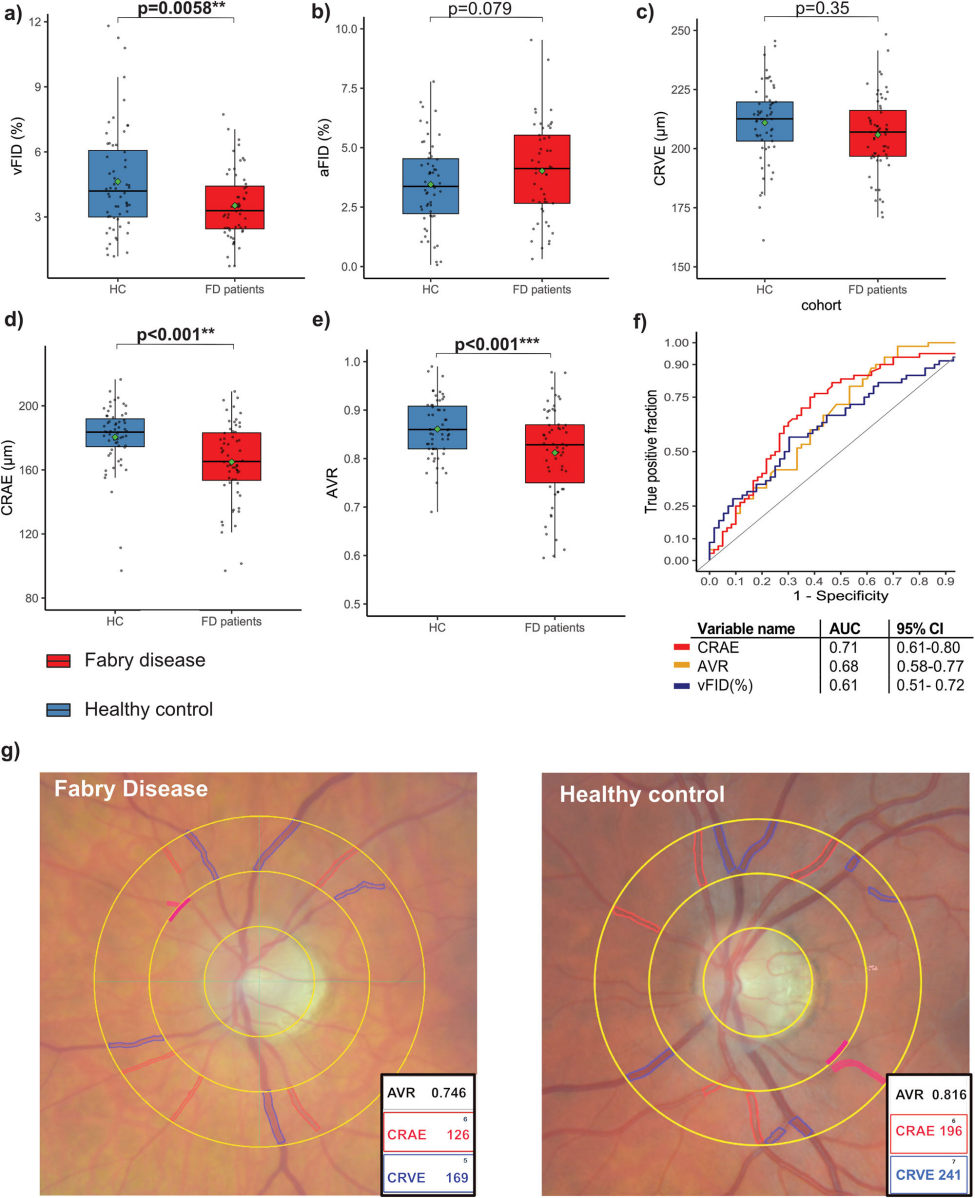
Dynamic retinal vessel and static retinal vessel parameters in Fabry disease patients compared to healthy controls*.* Violin plots of DVA parameters venular flicker-induced dilation (vFID: FD, n *=* 56; HC, n *=* 60; **a**) and arteriolar flicker-induced dilation (aFID: FD, n *=* 55; HC, n *=* 60; **b**) and SVA parameters CRVE; (**c**), CRAE; (**d**), and AVR; (**e** all n *=* 60) in age- and gender-matched Fabry patients (red) and healthy controls (blue). Boxplots display values as the median (line) and mean (green square). The Wilcoxon rank-sum test was used for non-parametric distributions, and the Student’s t-test was used for parametric distributions. Statistical significance is indicated as follows: *p *<* 0.05; **p *<* 0.01; ***p *<* 0.001. ROC curves for CRAE, AVR and vFID and their AUCs and 95% confidence intervals (CI) are shown (**f**). **g** shows representative fundus images of a Fabry disease patient and a healthy control, with semi-automatically labeled retinal arterioles and venules. Vessel calibers were measured one disc diameter away from the optic disc to derive the respective SVA parameters: CRAE, CRVE, and AVR. This figure was created with Affinity Publisher (Version 2.5.5).

FD patients demonstrated significantly lower venular flicker-induced dilation (vFID) compared to age- and gender-matched HC (vFID: 3.5% ± 1.6% vs. 4.6% ± 2.4%; p = 0.0058). No significant difference was observed in arteriolar flicker-induced dilation (aFID) between the groups, although FD patients showed a tendency towards higher values (3.9% ± 3.1% vs. 3.0% ± 2.0%; p = 0.079) (Fig. 1a, b). We analyzed retinal fundus pictures to assess retinal vessel diameters in FD patients. FD patients showed significantly narrower retinal arterioles (CRAE) when compared with HC (165.2 µm [153.5–183.2] vs. 183.2 µm [174.7–191.4], p < 0.001). There were no differences in retinal venular diameters (CRVE) between the groups (207.2 µm [196.9–217.2] vs. 212.6 µm [200.2–219.5]; p = 0.35). The arteriolar-venular ratio (AVR), calculated as CRAE/CRVE, was significantly lower in FD patients compared to HC (0.82 [0.74–0.87] vs. 0.86 [0.82–0.91]; p < 0.001) (Fig. 1c–e).

After controlling for potential confounders of RVA parameters, lower CRAE (p = 0.0028), lower AVR (p < 0.001), and lower vFID (p = 0.0015) remained significantly associated with FD (Supplementary Table 2). Additionally, vFID, CRAE, and AVR remained significantly lower in FD patients compared to HC after the exclusion of FD patients with a likely benign variant or VUS (Supplementary Fig. 1). CRAE had good accuracy in distinguishing FD patients from HCs, with an area under the curve (AUC) value of 0.71. Both vFID and AVR achieved acceptable discrimination with AUC values of 0.61 and 0.68, respectively (Fig. 1f). Representative fundus images of an FD patient and an HC are shown in Fig. 1g, illustrating the semi-automatic labeling of retinal arterioles and venules used to derive the SVA parameters CRAE, CRVE, and AVR.

**Fig. 2:**
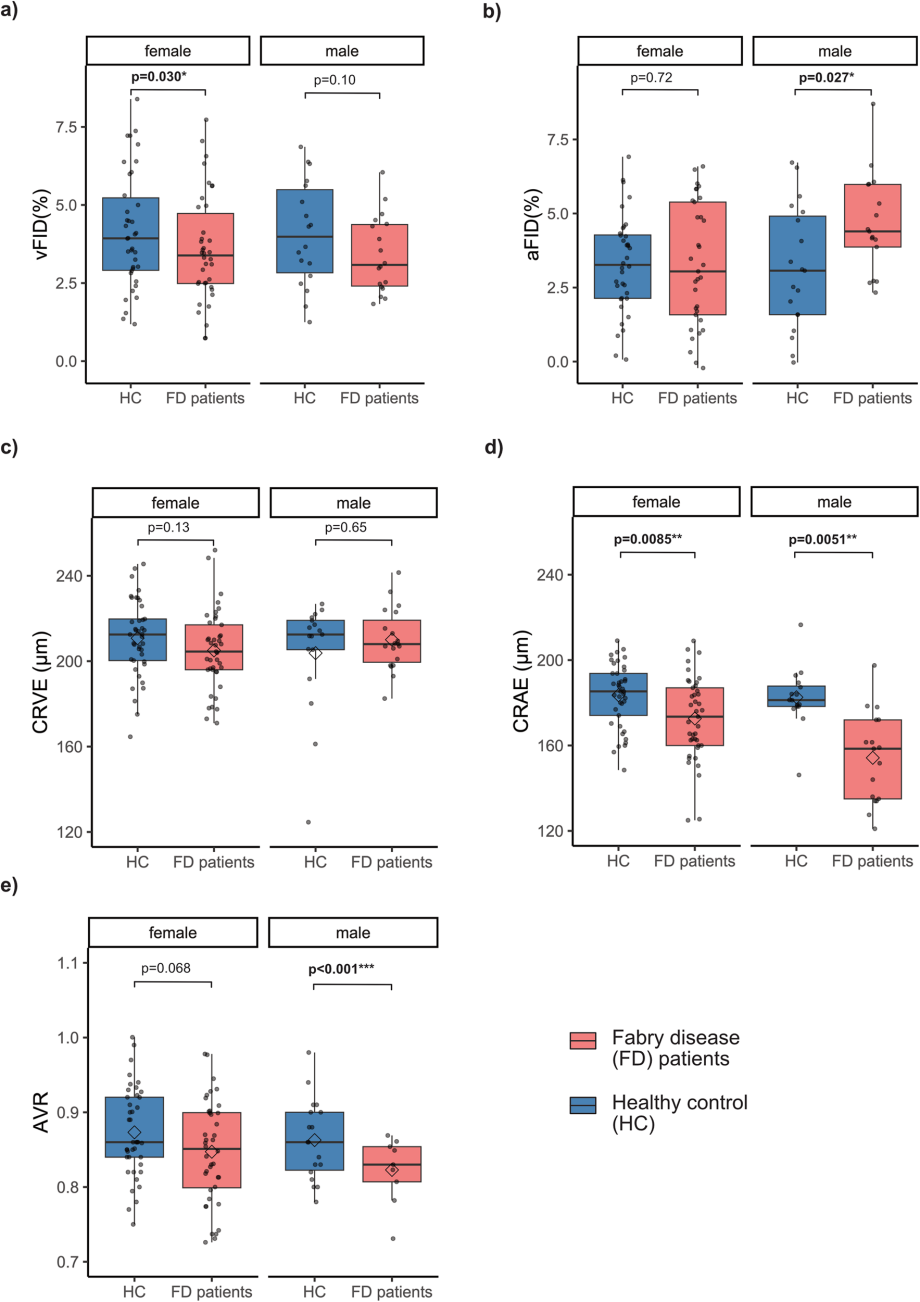
Dynamic and static retinal vessel parameters in male and female FD patients compared to HC after matching. Boxplots of DVA parameters, including vFID (**a**; FD: n = 56, HC: n = 60) and aFID (**b**; FD: n = 55, HC: n = 60), as well as SVA parameters CRVE, CRAE, and AVR (**c**–**e**; all n = 60), are shown for age- and sex-matched FD patients (red) and healthy controls (blue). These groups are subdivided into male (n = 19) and female (n = 41) patients. Boxplots display the mean (rectangle) and median (line). The Wilcoxon rank-sum test was applied for non-parametric distributions, and Student’s t-test was used for parametric distributions. Statistical significance is indicated as follows: *p < 0.05; **p < 0.01; ***p < 0.001. This figure was created with Affinity Publisher (Version 2.5.5).

FD is an X-linked inherited disease; therefore, we conducted a subgroup analysis. For DVA and SVA parameters, we saw similar trends for both genders. Female FD patients had lower vFID (p = 0.030) as did male patients, which, however, failed to reach significance (p = 0.10). Male patients exhibited a significant increase in aFID compared to HCs (p = 0.027), whereas no significant difference was found in female FD patients (p = 0.72) (Fig. 2a, b). CRVE did not differ significantly between female or male FD patients and HCs. CRAE and AVR were lower in male and female FD patients than HCs, with AVR not reaching significance (p = 0.068) in female patients (Fig. 2c–e). Thus, retinal microvascular changes are evident in male and female FD patients.

### Retinal vessel diameters and function as markers for clinical outcome in Fabry disease

**Fig. 3:**
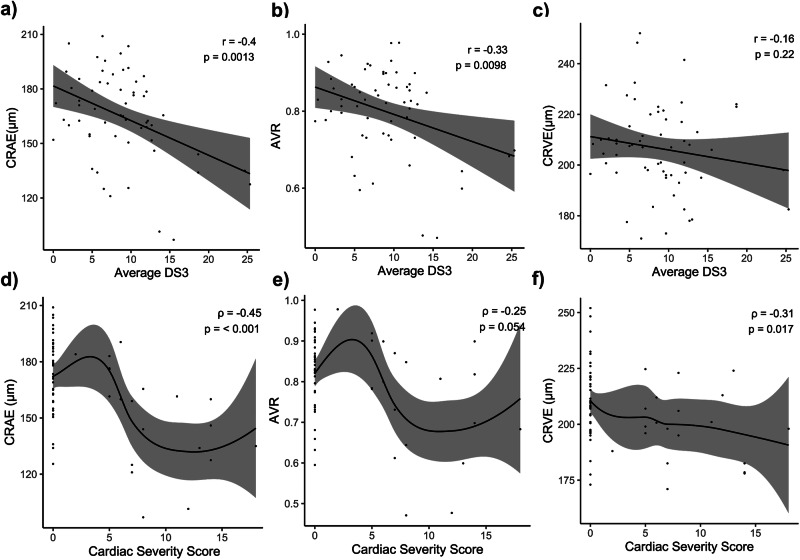
Correlation between static retinal vessel parameters with average DS3 and cardiac severity score. Scatterplots show the Pearson correlations between SVA parameters CRAE, CRVE, and AVR (all n=60) with average DS3 (n=63), including the corresponding correlation coefficient (r) and p-value (**a**–**c**). Spearman correlations are shown for CRAE, CRVE and AVR with the Cardiac Severity Score, including the corresponding correlation coefficient (ρ) LOESS curves with 95% confidence intervals are displayed to illustrate the negative, potentially non-linear associations (**d**–**f**). This figure was created with Affinity Publisher (Version 2.5.5).

Microvascular changes and their associated complications drive comorbidities and symptom severity in FD patients. Symptom severity in FD patients was assessed using the average DS3 score. CRAE showed a strong negative correlation with symptom severity (r = −0.40, p = 0.0013) (Fig. 3a). CRVE showed no significant correlation with average DS3 (r = −0.16, p = 0.22). However, lower AVR was significantly correlated with higher average DS3 (r = −0.33, p = 0.0098) (Fig. 3b, c). DVA parameters showed no association with average DS3 (not shown).

Among the four clinical domains of the average DS3, cardiac symptom severity was strongly correlated with narrower retinal arterioles (ρ = −0.45, p < 0.001) (Fig. 3d). Narrower retinal venules were significantly correlated with greater cardiac symptom severity (ρ = −0.31, p = 0.017), while the correlation for lower AVR missed significance (ρ = −0.25, p = 0.054) (Fig. 3e, f). As previously described by others, higher LysoGb3 levels were positively correlated with average DS3 and cardiac symptom (Supplementary Fig. 2a, b)^31^.

Multivariable linear regression models were fitted to further explore the association between the retinal microcirculation and the cardiac phenotype. Lower CRAE was significantly associated with higher ADS3, cardiac symptom severity and renal symptom severity, which all remained significant after adjusting for CV risk factors. Narrow retinal arterioles were independently linked to specific organ involvement, showing significant associations with LVH, HVD, CNVD, and CKD (Table 2). Similar findings were observed for higher LysoGb3, lower AVR and CRVE, which also showed significant associations with increased cardiac symptom severity and organ involvement (Supplementary Table 3).

**Table 2:** Associations between CRAE, disease severity scores and cardiac symptom burden

Predictor	Univariable		Mutlivariable	
	β-coefficient	*P* value	β-coefficient	*P* value
**Average DS3**	−0.40	0.0014**	−0.32	0.037*
**Cardiac Score**	−0.52	<0.001***	−0.46	<0.001***
**CNS Score**	−0.38	0.0031**	−0.29	0.054
**Renal Score**	−0.37	0.0035**	−0.29	0.030*
**CNVD**	−0.33	0.010*	−0.22	0.14
**LVH**	−0.42	<0.001***	−0.39	0.0034**
**HVD**	−0.41	0.0013**	−0.39	0.0028**
CHD	−0.24	0.063	−0.20	0.14
MI	−0.14	0.28	−0.12	0.36
HF	0.015	0.91	0.11	0.44
**CKD**	−0.43	<0.001***	−0.36	0.0091**

Linear regressions with the standardized β-Coefficient and the *p* value are shown for CRAE (*n* = 60) for different predictor variables. A multivariable model was fitted for potential confounders: arterial hypertension, hypercholesterolemia, nicotine abuse, and BMI. CNS central nervous system, CNVD central nervous vascular disease, LVH, left ventricular hypertrophy, HVD heart valve disease, CHD coronary heart disease, MI myocardial infarction, HF heart failure, CKD chronic kidney disease, LysoGb3 was log-transformed. CRAE as the dependent variable. Significant associations and values are marked in bold.

**Fig. 4:**
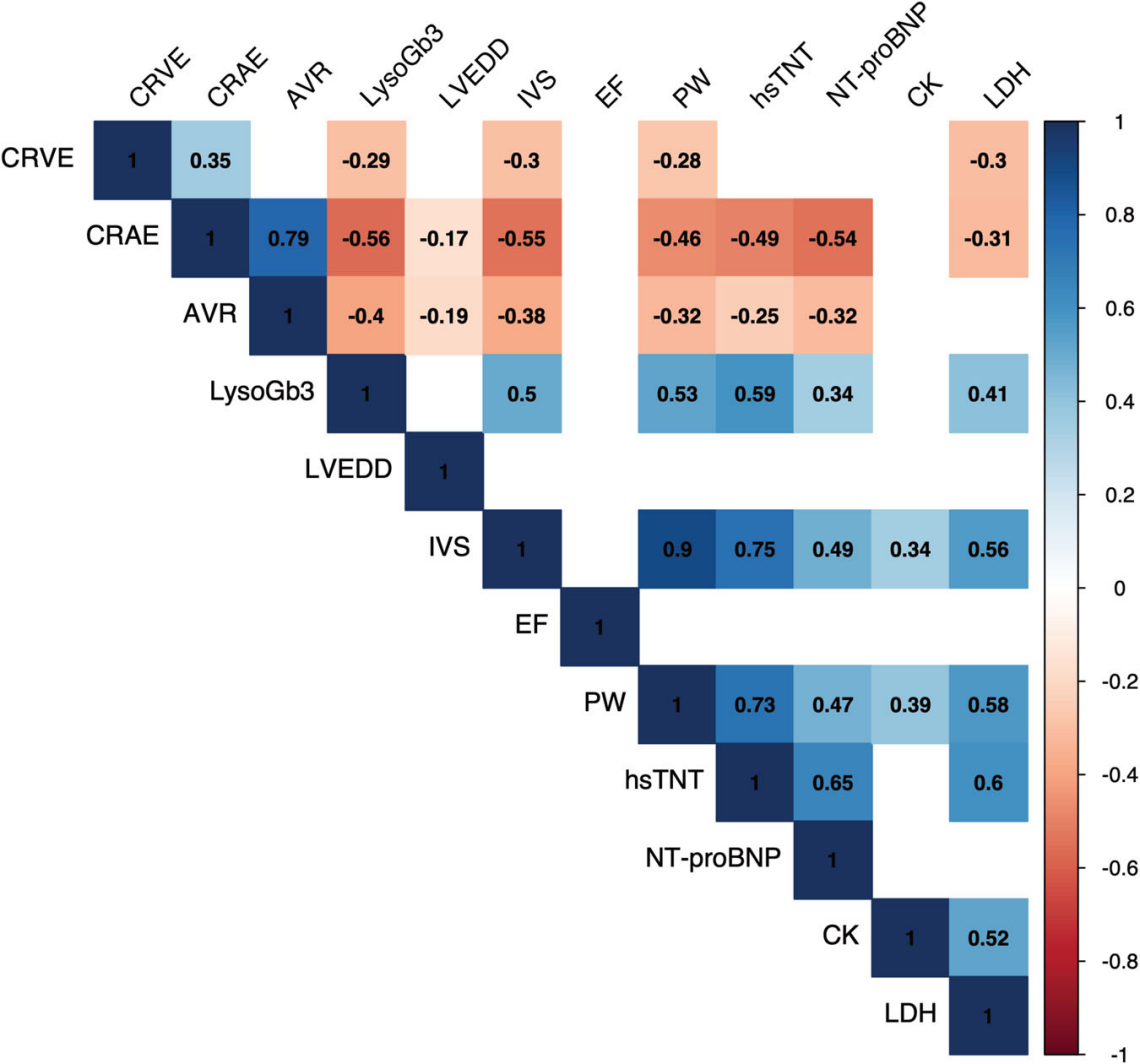
Correlations of static retinal vessel parameters and LysoGb3 with echocardiographic and laboratory parameters of cardiac damage. Correlation plots show Spearman correlations of static retinal vessel parameters and and LysoGb3 with cardiac laboratory parameters (hsTnT, high-sensitivity Troponin T, n=38; NT-proBNP, N-terminal prohormone of brain natriuretic peptide, n=38; CK, creatine kinase, n=63; LDH, lactate dehydrogenase, n=63) and echocardiographic parameters (LVEDD, left ventricular end-diastolic diameter, n=59; IVS, intraventricular septum thickness, n=60; PW, posterior wall thickness, n=58; EF, ejection fraction, n=46). Correlation coefficients (r) are visualized and displayed on the right. Non-significant correlations are not shown (empty fields)This figure was created with Affinity Publisher (Version 2.5.5).

Given that SVA parameters and higher LysoGb3 levels were linked to cardiac symptom scores and comorbidities, we further investigated how SVA parameters correlate with cardiac laboratory markers and echocardiographic parameters. Lower CRAE was significantly associated with elevated levels of lactate dehydrogenase (LDH, r = −0.31), high sensitivity Troponin T (hs-Troponin T, r = −0.49) and NT-proBNP (r = −0.54). Lower AVR showed similar results. Regarding echocardiographic changes, both CRAE and AVR were significantly negatively correlated with the left ventricular end-diastolic diameter (LVEDD, r = −0.17 and r = −0.19), intraventricular septum thickness (IVS, r = −0.55 and r = −0.38), and posterior wall thickness (PW, r = −0.46 and r = −0.32) (Fig. 4a, c). Except for the correlation with LVEDD, similar correlations were observed for LysoGb3 (Fig. 4b). Lower CRVE was correlated with higher IVS (r = −0.3), PW (r = −0.28), and LDH (r = −0.3) **(**Fig. 4d).

### Fabry genetic variants, cardiac involvement and associations with retinal microvascular health

Sequence variants were classified according to the American College of Medical Genetics and Genomics (ACMG) guidelines, and clinical parameters were compared among four variant categories: pathogenic (n = 33), likely pathogenic (n = 7), variants of uncertain significance (VUS, n = 8), and likely benign (n = 14). There was a trend towards a higher frequency of Fabry-related comorbidities in patients with pathogenic, likely pathogenic, and VUS, though this did not reach statistical significance. High sensitivity (hs)-CRP and hs-TnT levels were significantly different across these clusters, with higher hs-CRP values observed in patients with VUS and pathogenic variants and higher hs-TnT levels in patients with likely pathogenic and pathogenic variants (Supplementary Table 4).

Because left ventricular hypertrophy is one of the most frequent signs of Fabry-related cardiomyopathy, we analyzed the relationship between IVS thickness, variant classification, and specific GLA variants^32^.

In a multivariable linear regression model, IVS and LysoGb3 were significantly higher in VUS, likely pathogenic and pathogenic variants. Lower CRAE was associated with likely pathogenic and pathogenic variants (Supplementary Table 5).

Next, we assessed the relationship between specific GLA-gene sequence variants and IVS thickness in a multivariable model. Seven gene variants with a potential cardiac phenotype were selected based on their estimates and significant association with higher IVS, suggesting their relevance in FD-related cardiac hypertrophy (Supplementary Table 6).

**Fig. 5:**
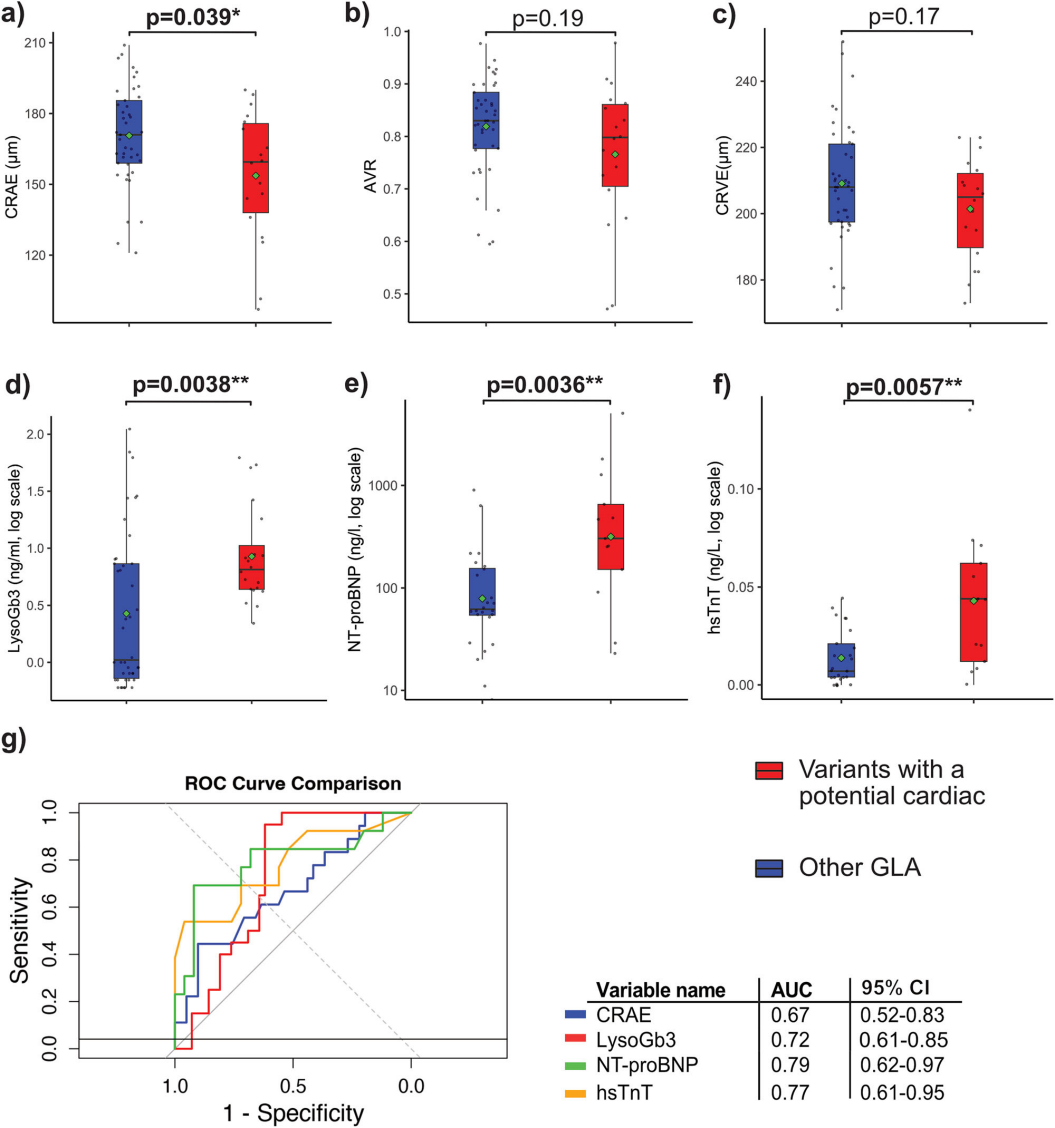
Static retinal vessel parameters, LysoGb3 and cardiac laboratory values in FD patients with and without a potential cardiac GLA gene variant*.* Boxplot of CRAE (**a**), AVR (**b**) and CRVE (**c**; all n = 60), LysoGb3 (**d**; n = 63), and laboratory parameters NT-proBNP (**e**; n = 38) and hsTnT (**f**; n = 38) between FD patients with a potential cardiac variant (red, n = 20) and FD patients with other GLA gene variants (blue, n = 43). LysoGb3, hsTnT and NT-proBNP were log-transformed. Boxplot displays values as median (line) and mean (green square). The Wilcoxon rank-sum test was used for non-parametric distributions, and the Student´s t-test was used for parametric distributions. * p < 0.05; **p < 0.01; ***p < 0.001. ROC curves for distinguishing gene variants are shown for CRAE, LysoGb3, NT-proBNP and hsTnT and their AUCs and 95% confidence intervals (CI) are shown (**g**).

We plotted a heatmap of scaled estimates for various cardiac outcome variables to further visualize potential cardiovascular involvement in the seven selected gene variants. Except for one variant (c.547 G > A), all selected gene variants showed associations with either levels of higher cardiac laboratory parameters (LDH, CK, NT-proBNP, hsTnT) or higher LVEDD and PW thickness (Supplementary Fig. 3). Based on these associations across multiple cardiac parameters, six variants were finally classified as “potential cardiac variants”. Next, we compared parameters of retinal microcirculation and LysoGb3 between FD patients with the six selected variants with a potential cardiac phenotype (n = 20) and FD patients with other GLA-gene sequence variants (n = 42). FD patients with a potential cardiac phenotype showed a significant increase in LysoGb3 levels (log-transformed, 0.81 vs. 0.02, p = 0.0038). Additionally, they showed significantly narrower retinal arterioles (159.5 µm vs. 171.0 µm, p = 0.039). Both CRVE and AVR tended to lower levels, but not significantly (Fig. 5a–d). Showcasing cardiac involvement, NT-proBNP, and hsTnT were significantly higher in the selected six gene variants (Fig. 5e, f). LysoGb3, hsTnT and NT-proBNP had good accuracy in distinguishing FD patients with a cardiac variant from patients without, with AUC values of 0.72, 0.77 and 0.79. CRAE achieved acceptable discrimination with an AUC value of 0.67 (Fig. 5g). Four of the variants with a potential cardiac phenotype were pathogenic, one likely pathogenic, and interestingly, one was a VUS. The selected gene variants were prominent in female FD patients (female 15/20 vs. male 5/20).

### Inflammatory parameters and markers of endothelial dysfunction in FD patients

**Fig. 6:**
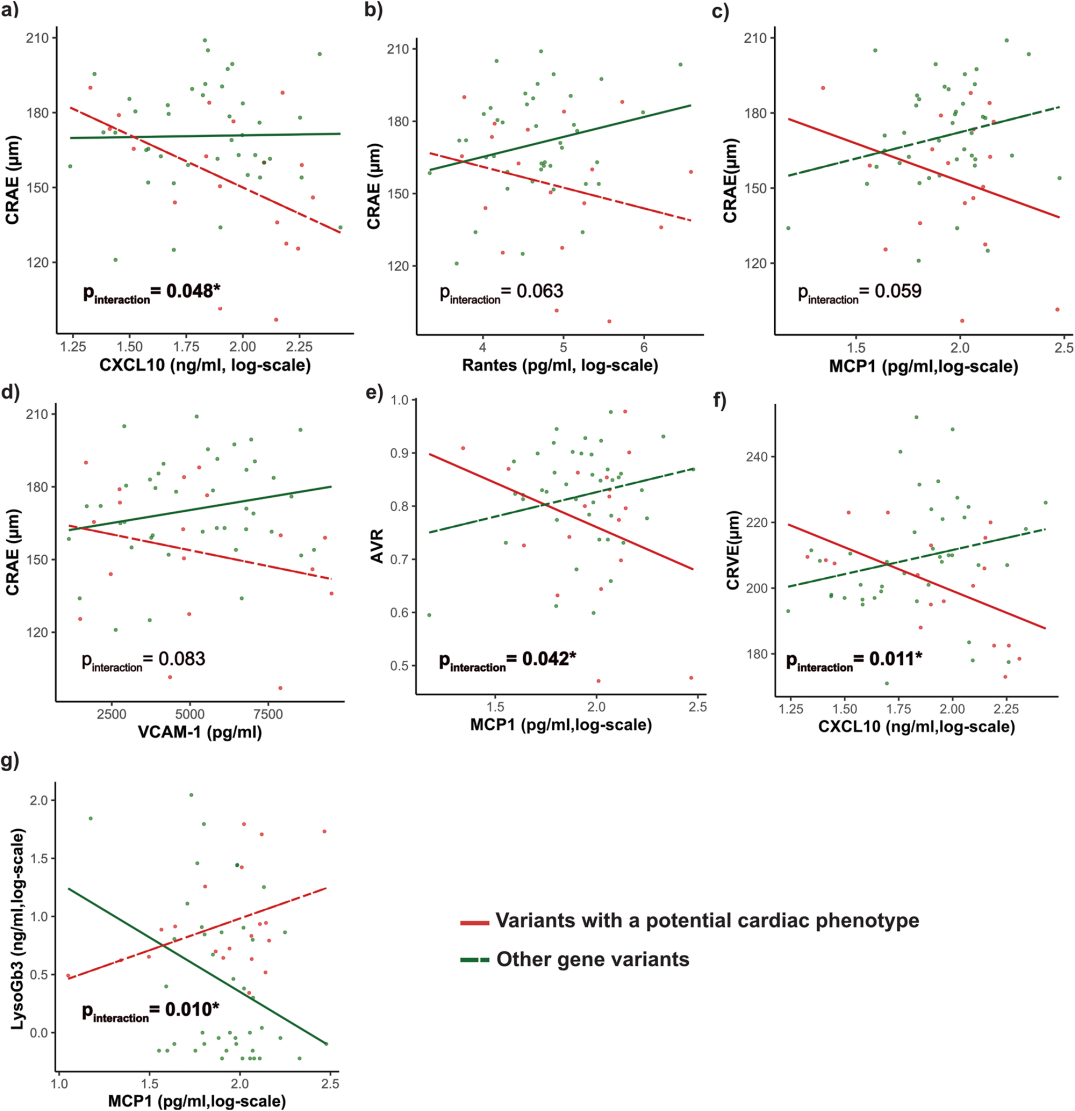
Interaction of endothelial dysfunction and inflammatory markers with static retinal vessel parameters and LysoGb3 in FD patients with a potential cardiac GLA gene variant. Interaction plots visualize the effect of cardiac gene variants (red line, n = 20) and no cardiac variants (green, dashed line, n = 42) on the correlation between CRAE (**a**–**d**), AVR (**e**), CRVE (**f**), and LysoGB3 (**g**) with levels of inflammatory and endothelial dysfunction parameters. MCP1, CXCL10, Rantes and LysoGb3 were log-transformed (all n = 63). P_interaction_ displays the interactions between FD patients with a potential cardiac gene variant and laboratory variables calculated in a multivariable linear model. This figure was created with Affinity Publisher (Version 2.5.5).

Previous studies have reported elevated markers of endothelial activation and chronic inflammation in FD patients. Compared to age- and gender-matched HC, FD patients displayed significantly elevated levels of VEGF (pg/ml, 67.3 [39.5–118.0] vs. 0.7 [0.0–2.1], p < 0.001), ICAM-1 (ng/ml, 159.5 [78.6–333.6] vs. 0.0 [0.0–244.1], p = 0.039) and VCAM-1 (ng/ml, 48.9 [30.9–68.0] vs. 0 [0.0–25.2], p = 0.0011). In addition, levels of CXCL10 (pg/ml, 79.3 [45.9–120.3] vs. 37.5 [29.2–59.6], p < 0.001), MCP1 (pg/ml, 94.9 [63.4– 117.7] vs. 37.0 [26.2– 65.0], p < 0.001) and Rantes (ng/ml, 50.1 [15.0–125.6] vs. 1.4 [0.8–1.6], p < 0.001) were elevated. IL-6 showed a tendency towards higher levels, however failing to reach significance (Supplementary Fig. 4a–f). We fitted regression models with interaction effects to investigate the association of markers of ED and inflammation markers with retinal vessel diameters and function in FD patients with a potential cardiac gene variant (Supplementary Table 7). In FD patients with potential cardiac variants, the association between higher levels of CXCL10 and lower CRAE was stronger, with a significant interaction observed between CXCL10 and cardiac variants (p_interaction_ = 0.048) (Fig. 6a). Similar trends were found in FD patients with cardiac variants for the association between higher levels of MCP1, Rantes, and VCAM-1 and lower CRAE, although these interactions failed to reach significance (Fig. 6b–d). Consistent with these findings, in FD patients with a potential cardiac variant, we observed a stronger association between higher CXCL10 levels and lower CRVE (p_interaction_= 0.011) as well as between higher MCP1 levels and lower AVR (p_interaction_= 0.041) (Fig. 6e, f). Interestingly, higher levels of LysoGb3 were associated with higher MCP1 in FD patients with potential cardiac variants (p_interaction_= 0.010) (Fig. 6g).

## Discussion

Endothelial dysfunction is a hallmark of Fabry disease, and cardiac impairment such as perfusion defects and slow coronary flow result from luminal narrowing in the microcirculation^10,23,33^. For the first time we could now show an altered retinal microcirculation in FD patients, as evidenced by significantly reduced flickering-induced venular dilatation, retinal arteriolar narrowing, and smaller AVR. Importantly, these microvascular differences were independent of age, gender, and CV risk factors such as arterial hypertension, BMI, hypercholesterolemia, and smoking.

Smooth muscle cell (SMC) proliferation is one of the consequences of Gb3 deposition, and SMC proliferation is directly induced by LysoGb3^21,34^. As small arteries and arterioles in the retina are known to have relatively large components of SMCs, arteriolar narrowing, as evidenced by smaller CRAE in our FD patients, might be a direct effect of intima thickening^25^. SMC proliferation may lead to remodeling of the arterioles, increasing shear stress, which ultimately reduces NO synthesis. Studies also postulate a downregulation of the NO signaling pathway in FD^35,36^. Both could explain our findings of reduced flicker-induced venular dilation. As we have shown that reduced vFID is an independent predictor of mortality in a cohort of patients with end-stage renal disease (ERDS), assessing venular dilation in FD patients could also provide valuable predictive insights, particularly in identifying patients at higher risk of Fabry-associated complications, such as cardiac or cerebrovascular events^29^. Systemic, ongoing inflammation may serve as the unifying pathophysiological mechanism underlying reduced venular dilation in both conditions^3,37^.

Interestingly, in male FD patients, flickering-induced arteriolar dilation was significantly higher than in healthy males. This might be explained by a compensatory overregulation in arterioles and, therefore, a stronger reaction to flickering light. Altarescu et al. also showed a stronger arterial vasodilation in FD patients after administration of acetylcholine^35^. This is in line with findings in Alzheimer’s disease (AD), where the levels of storage products directly correlated with a higher arteriolar pulse amplitude, and the arteriolar pulse amplitude was significantly higher in AD patients^38,39^.

In female FD patients, CRAE and vFID were significantly reduced compared to HC, indicating that RVA provides a tool to quantify microcirculatory changes in female patients. In patients with residual enzyme activity, such as female patients or those with cardiac variants, SMCs showed deposits even when the endothelium remained clear. Therefore, SMCs may be more susceptible to Gb3 than the endothelium^40^. Monitoring female FD patients with RVA could provide additional insights into endothelial health beyond measuring often normal LysoGb3 levels. We argue that RVA could offer a cost-effective, non-invasive tool to monitor microvascular endothelial health in FD patients and, therefore, should be incorporated into routine care. Although a retinal phenotype was detectable in both genders, the more pronounced retinal microvascular alterations observed in male Fabry patients likely reflect the X-linked inheritance of the disease, resulting in a higher accumulation of storage products and a more severe vascular phenotype in males compared with females^21,41,42^. Although a higher cardiovascular burden in males or age-related factors could theoretically contribute to these differences, both cardiovascular risk profiles and age were comparable between genders in our cohort (see Supplementary Table 1). Therefore, the X-linked nature of the disease likely represents the principal explanation for the observed gender-related differences in retinal microvascular health.

Whether ERT effectively clears Gb3 deposits in the heart remains controversial, and microvascular changes may persist well beyond therapy^18,24,43^. In patients with more advanced disease, ERT’s impact on restoring vascular health is potentially limited or altered, possibly due to longstanding disease-related vascular changes that are less responsive to ERT^6,44^. One possible explanation could be persistent alterations in endothelial signaling pathways^17,45^.

Due to the increased availability of genetic testing and disease awareness, there is an evolving number of VUS^46^. Determining whether to start ERT can be challenging, especially with late-onset or organ-specific variants, such as cardiac ones^8,47^. Although pathogenic variants tended to be associated with more Fabry-related complications in our cohort, complications were also observed in cases with VUS. After identifying six variants closely associated with primarily cardiac involvement during recruitment and predominantly found in female FD patients, we observed that these variants are strongly linked to arterial narrowing. This finding aligns with our observation that retinal arteriolar narrowing is closely associated with cardiac comorbidities, such as LVH, HVD, and CNVD, independent of traditional cardiovascular risk factors. Barbey et al. showed a proliferative effect of plasma from FD patients on SMC and cardiomyocytes, suggesting the plasma contains factors sustaining proliferative activity^48^. In our cohort, the FD plasma showed elevated levels of chemokines, such as Rantes, MCP1, and CXCL10.

Rantes and MCP1 are upregulated in human podocytes after Lyso-Gb3 stimulation by the NOTCH-1-dependent pathway, contributing to podocyte injury and kidney fibrosis^49^. Additionally, in light of evidence linking the CXCL10/CXCR3 axis to proinflammatory and T-cell-mediated responses in cardiac diseases, our finding of elevated CXCL10 in patients may suggest a potential role for CXCL10-mediated inflammation in the pathogenesis of cardiac complications in this cohort^50^. This, aligns with the findings of Frustaci et al., who reported immune-mediated myocarditis in Fabry cardiomyopathy characterized by CD3 + T-cell infiltration and myocyte necrosis^51^. Chemokine-driven immune cell recruitment and amplified inflammatory signaling in endothelial cells may upregulate markers of endothelial activation in FD patients. We could show significantly higher levels of VEGF, ICAM-1, and VCAM-1. ICAM-1 and VCAM-1 are upregulated in EC after Gb3 stimulation, and VEGF has been associated with hypertrophic cardiomyopathy in FD patients^13,52^. In FD patients with potential cardiac variants, we could show that higher levels of MCP1 and CXLC10 are associated with an impaired retinal microcirculation and higher levels of LysoGb3. Cardiac inflammation and endothelial activation are secondary mechanisms sustaining damage to myocardial structures despite ERT^2,3^. Higher levels of autoantibodies in FD patients further point toward a shared pathophysiology of autoinflammatory diseases and FD^53^. This further emphasizes how crucial early detection of microvascular changes in FD is and suggests that concomitant anti-inflammatory therapies should be considered in advanced disease.

This study is cross-sectional; therefore, even though we tried to control for confounders, all correlations and associations should be interpreted as exploratory. Longitudinal studies are needed to confirm the predictive value of RVA parameters. While the study includes a relatively large cohort of 63 FD patients, which is significant given the rarity of the disease, subgroup analyses may still lack statistical power to detect certain associations. Furthermore, our cohort consisted primarily of heterozygous patients, and we included FD patients with VUS and likely benign variants. While subgroup analysis was performed, this composition may limit the generalizability of the findings. In addition, all patients were recruited from the Department of Nephrology, which may introduce a selection bias toward individuals with renal involvement. However, each participant underwent comprehensive multidisciplinary evaluations by a nephrologist, ophthalmologist, neurologist, and cardiologist to ensure systematic assessment of multi-organ manifestations.

Regarding the markers analyzed as secondary outcomes, it is essential to note that while the study was sufficiently powered to assess the primary outcome of endothelial dysfunction, the exploratory analyses involving laboratory markers and associations with genetic variants may not have been adequately powered to detect all possible associations. The results of these secondary analyses should be interpreted with caution and viewed as hypothesis-generating, providing a foundation for further studies with larger cohorts to validate these findings. Despite these limitations, this study represents a meaningful contribution to understanding endothelial dysfunction and its potential biomarkers in a rare disease like Fabry. Our study highlights the value of RVA as a quick, inexpensive, and validated tool for early detection of microvascular changes in Fabry disease, independent of traditional cardiovascular risk factors. The significant alterations observed in both static and dynamic retinal vessel parameters in male and female FD patients underline the close link between microvascular changes and systemic disease severity. Notably, the strong correlation between retinal arteriolar narrowing and established echocardiographic and laboratory markers of cardiovascular involvement suggests that RVA may be a reliable, non-invasive approach for monitoring endothelial health in Fabry disease patients.

Given the persistent inflammatory and endothelial activation seen in FD, particularly in patients with a potential cardiac gene variant, early detection and monitoring of microvascular abnormalities through RVA could enable more timely intervention and the possibility of incorporating adjunctive anti-inflammatory therapies to slow disease progression. Longitudinal studies are essential to assess the predictive value of RVA for long-term cardiac- and Fabry-related outcome variables.

## Methods

### Study design and patients

This study is part of an observational longitudinal investigation examining the retinal microvasculature of 63 FD patients and providing an in-depth clinical characterization of FD patients. All patients were recruited in our outpatient clinic from the department of nephrology (Klinikum Rechts Der Isar, Technical University of Munich, School of Medicine), which all underwent clinical examination, RVA analysis, and blood collection. The local ethics committee approved the study protocol (Ethics Committee of the Klinikum Rechts Der Isar, Technical University of Munich, School of Medicine, No. 432/19S). The study “VASCinFABRY” was registered previously at ClinicalTrials.gov (NCT06758648). All participants of this study gave written informed consent. The study conforms to the principles of the Declaration of Helsinki and was designed following the Strengthening the Reporting of Observational Studies in Epidemiology (STROBE) guidelines. The sample size was defined previously in the study protocol and was powered for the primary aim of comparing retinal vessel parameters in Fabry patients with a healthy cohort (HC). Assumptions regarding expected differences and variability were informed by comparable patient cohorts, and the calculation was conducted with guidance from a statistical expert. The HC was recruited as described previously^30^.

Inclusion criteria were age >18, diagnosis of FD by genetic testing of the galactosidase alpha (GLA) gene, α-gal A activity in leukocytes, or elevated LysoGb3 levels. Exclusion criteria were active infection and or malignant disease, operation two weeks before baseline examination, known glaucoma or epilepsy, and lack of written consent.

Genetic analysis was done in 62 patients (Genetic analysis, Molecular Genetics and Metabolism Laboratory, Centogene AG, Rostock, Germany).

### Retinal vessel analysis

DVA and SVA were performed by experienced and trained examiners using the Dynamic Retinal Vessel Analyzer (DVAlight; IMEDOS Systems, Jena, Germany) and the Static Vessel Analyzer (IMEDOS Systems GmbH, Jena, Germany). The technique has been extensively described in our studies^29,54^. SVA was performed before DVA. Before the examination, pupils were dilated using topical tropicamide (0.5% Mydriaticum Stulln; Pharma Stulln, Germany), and patients were seated in a quiet, dark room for a ten-minute rest period.

In the case of DVA, patients were asked to focus on a needle attached inside the camera, and the diameters of one arteriole and venule were automatically and continuously recorded for 350 seconds. Arteriole and venule segments between 0.5 to 1 mm were analyzed approximately one disc diameter away from the optic nerve in the upper-temporal or lower-temporal direction. The baseline recording was 50 seconds, followed by a flickering phase of 20 seconds and then a recovery period of 80 seconds. Three of these cycles were performed, and we calculated the percentage of maximum arteriolar (aFID) and venular dilation (vFID) to baseline as previously described. For data validation, the quality of vessel response curves was compared using a cumulative scoring method (range 0 to 5)^29^. Retinal measurements with a score <2.5 were excluded after re-evaluation by an experienced observer. In five cases (7.9%), we could not measure vFID due to a lack of information in the measured region, and in six cases (9.5%), we could not obtain high-quality measurements of aFID.

SVA pictures were analyzed using Vesselmap 2^®^ (IMEDOS Systems GmbH, Jena, Germany). One eye was examined, and three images were taken with the focus on the optic disc at an angle of 50°. Roughly one disc diameter away from the optic disc, retinal veins and arterioles segments were semi-automatically labeled. The Paar-Hubbard formula averaged the central retinal arteriolar (CRAE) and central venular (CRVE) equivalents^55^. SVA parameters CRAE and CRVE were measured in measurement units (MU), where 1 MU corresponds to 1 µm in Gullstrand’s eye model. Inter- and intra-observer inter-class correlation coefficients for CRAE and CRVE range from 0.75 to 0.87^55,56^. In three patients (4.8%), we could not obtain high-quality static retinal pictures. For both manual and semi-automated image analyses, the examiner was aware that the analyzed cohort consisted of patients with Fabry Disease. However, to minimize potential bias, the examiner was blinded to all individual clinical information, including disease severity, biomarker levels (e.g., LysoGb3), echocardiographic parameters, and genetic data during image evaluation.

### Analysis of clinical and laboratory characteristics

We studied the medical records to record baseline characteristics, comorbidities, disease onset, gene variants, FD-specific treatment, and medication. Blood examinations and echocardiography took place within the framework of routine tests on the day of baseline recruitment.

Blood samples were collected following previously described protocols^54^, and routine parameters were measured in an ISO-certified laboratory. Levels of IL-6, Rantes, VEGF, CXCL10, MCP1, VCAM-1, and ICAM-1 in patient serum were quantified using the Cytometric Bead Array Flex system (BD Biosciences, San Diego, USA), following the manufacturer’s instructions.

The Fabry disease severity scoring system (average DS3) is a validated score that takes four clinical domains (Renal, peripheral nervous system, cardiac, and central nervous system) and one patient-reported outcome (PROM) into account. The average scoring system ranges from “minimal disease severity” (0 p.) to “maximum disease severity” (32 p.)^57^. Interventricular-septum (IVS) thickness was measured and considered as increased for values > 13 mm. The presence of chronic kidney disease (CKD) was defined as an eGFR of less than 60 ml/min calculated with the CKD-EPI equitation.

In 62 patients, the galactosidase alpha (GLA)-gene sequence variants were analyzed. Variants were classified according to the American College of Medical Genetics and Genomics (ACMG) guidelines, and 33 patients were classified with a pathogenic variant, 7 with a likely pathogenic variant, 8 patients with a variant of uncertain significance (VUS) and 14 with a likely benign variant (Supplementary Table 8)^58^.

### Statistical analysis

All statistical analyses were performed using R (4.2.1) and R Studio (Version: 2024.04.2 + 764). Graphical preparation was done using Affinity Publisher (Version 2.5.5). The Graphical Abstract was Created with BioRender.com. Normally distributed values are shown as mean, ± standard deviation (SD), and non-normally distributed values as mean and their interquartile range (IQR) if not otherwise stated. To compare patients’ characteristics student´s t-test was used for normally distributed values, the Wilcoxon rank sum test for non-normally, and χ^2^-test for categorical variables. For comparisons of more than two groups, one-way ANOVA was used for normally distributed data, and Kruskal-Wallis was used elsewhere. Normality was assessed with the Shapiro-Wilks test and visually by using histograms. All statistical hypothesis testing was conducted on two-sided 5% significance levels. To match FD patients with the healthy cohort based on age and gender, we used the Matching package; matching success was controlled with the MatchBalance function. Violin- and boxplots were created using the ggplot package. Receiver operating characteristics (ROC) analysis was done with the plotROC and pROC package, and the area under the curve (AUC) was calculated using the trapezoidal rule. The 95% confidence interval (CI) of the AUC was calculated using DeLong´s method. For the correlation plots and the calculation of Spearman´s (for skewed data) and Pearson´s (for normal data) correlation coefficients, we used the ggscatter function, which is part of the ggpubr package. The correlation heatmap was created using the corplot package. In a multivariable linear model, we adjusted for potential confounders (BMI, arterial hypertension, hypercholesterolemia, and nicotine abuse) of retinal vessel analysis^59–62^. We used the interaction package to create interaction plots. Two independent researchers typed in all clinical data for double-data verification. Efforts were made to minimize missing data throughout the study, and the extent of missingness was kept to a reasonably achievable minimum. Missing data were not imputed. One author had full access to all the data in the study and took responsibility for its integrity and the data analysis.

## Supplementary information

Supplementary Information

Transparent Peer Review file

## Data Availability

Data are available from the corresponding author at reasonable request.
