# Supplementary material for: Endothelial dysfunction in Fabry disease: retinal biomarkers link cardiac GLA gene variants with chronic inflammation
Source: NPJ Genom Med. 2026 Jan 16;11:6. doi: 10.1038/s41525-025-00540-1 (PMC12824269; doi:10.1038/s41525-025-00540-1)
Supplement: Supplementary file 1 — Supplementary Information [file 41525_2025_540_MOESM1_ESM.pdf]

Supplementary material

Endothelial Dysfunction in Fabry Disease: Retinal Biomarkers Link Cardiac GLA Gene Variants with Chronic Inflammation

Timon Wallraven[*] and Claudia Regenbogen[1*], Roman Günthner[1], Andrea Riberio[1], Javier Carbajo-Lozoya [1], Nora Hannane[1], Michael Wunderle[1], Abdalrahman Assaf[1], Maciej Lech[2], Henner Hanssen[3], Lukas Streese[3], Derralynn Hughes[4], Bernhard Haller[5], Konstantin Kotliar[6], Uwe Heemann[1], Christoph Schmaderer[1,7]

[1]TUM School of Medicine and Health, Department of Nephrology, TUM University Hospital, Technical University of Munich, Munich, Germany

[2]Medizinische Klinik Und Poliklinik IV, LMU University Hospital Munich, Munich, Germany

[3]Department of Sport, Exercise and Health, Preventive Sports Medicine and Systems Physiology, University of Basel, Basel, Switzerland

[4]Lysosomal Storage Disorders Unit, The Royal Free London NHS Foundation Trust and University College London United Kingdom

[5]TUM School of Medicine and Health, Institute of AI and Informatics in Medicine, TUM University Hospital, Technical University of Munich, Munich, Germany

[6]Aachen University of Applied Sciences, Heinrich-Mussmann-Str. 1, 52428, Jülich, Germany

[7]German Centre for Infection Research (DZIF), Partner Site Munich, Munich, Germany

***These authors contributed equally to this work**

**Clinical Characteristics**	**Female (n=43)**	**Male (n=20)**	***P* value**
**Age**			
y, Mean(SD)	47.4 (±18.2)	50.4 (±10.7)	0.51
**BMI**			
kg/m^2, Median (IQR)	23.9 (21.8 - 27.9)	24.1 (20.9 – 25.4)	0.45
Cardivascular risk factors			
**Hypercholesterolemia**	20 (46.5%)	14 (70.0%)	0.17
**Arterial hypertension**	15 (34.8%)	6 (30.0%)	0.78
**Nicotine abuse**	8 (18.6%)	9 (45.0%)	0.078
Fabry-related complications			
**LVH**	16 (37.2%)	14 (70.0%)	**0.029***
**Cardiac arrhythmia**	9 (20.9%)	6 (30.0%)	0.53
**HVD**	0 (0.0%)	3 (15.0%)	**0.028***
**CHD**	2 (4.7%)	3 (15.0%)	0.32
**CNVD**	6 (13.9%)	11 (55.0%)	**0.0011****
**CKD**	1 (2.3%)	3 (15.0%)	0.15
Fabry disease severity and treatment			
**ERT or PCT**	30 (69.7%)	13 (65.0%)	0.77
**Therapy duration**			
y, Median (IQR)	1.1 (0.0 – 1.9)	0.6 (0.0 – 15.2)	0.46
**Average DS3**			
Median (IQR)	7.7 (4.7 - 9.7)	11.7 (7.2 - 14.1)	**0.0052****
**LysoGb3 (ng/ml)**			
ng/ml, Median (IQR)	4.2 (0.9 - 6.9)	27.0 (0.9 - 51.6)	**0.028***
Laboratory parameters			
**Creatinine**			
mg/dl, Median (IQR)	0.8 (0.7 - 0.9)	1.1 (0.9 - 1.2)	**< 0.001*****
**Ferritin**			
µg/l, Median (IQR)	75.0 (56.0 - 113.0)	213.0 (162.0 - 256.0)	**< 0.001*****
**Leukocytes**			
G/l, Median (IQR)	6.9 (5.4 – 7.5)	6.3 (5.1 – 6.7)	0.44
**hsCRP**			
mg/l, Median (IQR)	0.11 (0.07- 0.21)	0.09 (0.07 – 0.14)	0.96
**hsTnT**			
ng/l, Median (IQR)	0.01 (0.002- 0.03)	0.02 (0.007 – 0.04)	0.17
**NT-proBNP**			
pg/ml, Median (IQR)	112.0 (58.8 – 267.8)	60.0 (34.8 – 206.8)	0.48

Supplementary Table 1 Baseline Characteristics of male and female FD patients P-values are reported for statistical tests comparing male (n=20) and female (n=43) FD patients. Student's t-test was used for parametric variables, the χ^2 test for categorical variables, the Wilcoxon rank-sum test for nonparametric variables, and Fisher's exact test for proportional variables. The abbreviations used are as follows: BMI, body mass index; LVH, left ventricular hypertrophy (intraventricular thickness > 13 mm); HVD, heart valve disease; CHD, coronary heart disease; CNVD, central nervous vascular disease (including TIA and stroke); CKD, chronic kidney disease (GFR < 60 ml/min/1.73 m^2); ERT, enzyme replacement therapy; PCT, pharmacological chaperone therapy; LysoGb3 (n=63); Creatinine (n=63); Ferritin (n=63); Leukocytes (n=63); hsCRP, high-sensitivity C-reactive protein (n=56); hsTnT, high-sensitivity troponin T (n=38); NT-proBNP, N-terminal prohormone of brain natriuretic peptide (n=38). Statistical significance is indicated as follows: *p < 0.05, **p < 0.01, ***p < 0.001.

vFID as the dependent variable

Predictors	Univariate		Multivariable	
	ß-Coefficient	*P-value*	ß-Coefficient	*P-value*
Age, year	0.048	0.61	-	-
Sex, male	0.041	0.66	-	-
Art. hypertension	0.11	0.24	-	-
Nicotine abuse	-0.041	0.68	-	-
BMI	0.073	0.46	-	-
**Fabry Disease**	**-0.26**	**0.0050****	**-0.33**	**0.0015****

CRAE as the dependent variable

Predictors	Univariate		Multivariable	
	ß-Coefficient	*P-value*	ß-Coefficient	*P-value*
**Age, year**	**-0.27**	**0.0029****	-	-
**Sex, male**	**-0.36**	**<0.001*****	-	-
**Art. hypertension**	**-0.24**	**0.0078****	-	-
Nicotine abuse	-0.018	0.85	-	-
BMI	0.035	0.71	-	-
**Fabry Disease**	**-0.27**	**0.0028****	**-0.28**	**0.0028****

AVR as the dependent variable

Predictors	Univariate		Multivariable	
	ß-Coefficient	*P-value*	ß-Coefficient	*P-value*
**Age, year**	**-0.19**	**0.041***	-	-
**Sex, male**	**-0.38**	**<0.001*****	-	-
Art. hypertension	-0.084	0.36	-	-
Nicotine abuse	-0.11	0.22	-	-
BMI	0.0093	0.92	-	-
**Fabry Disease**	**-0.36**	**<0.001*****	**-0.39**	**<0.001*****

Supplementary Table 2 Associations of vFID, CRAE and AVR with Fabry Disease after controlling for potential confounders Linear regressions with standardized β-coefficients and p-values are shown for CRAE (n=60), AVR (n=60), and vFID (n=56) across different variables. A multivariable model was fitted to adjust for potential confounders, including age, sex, arterial hypertension, nicotine abuse, and BMI. Statistical significance is indicated as follows: $*p < 0.05$; $**p < 0.01$; $***p < 0.001$.

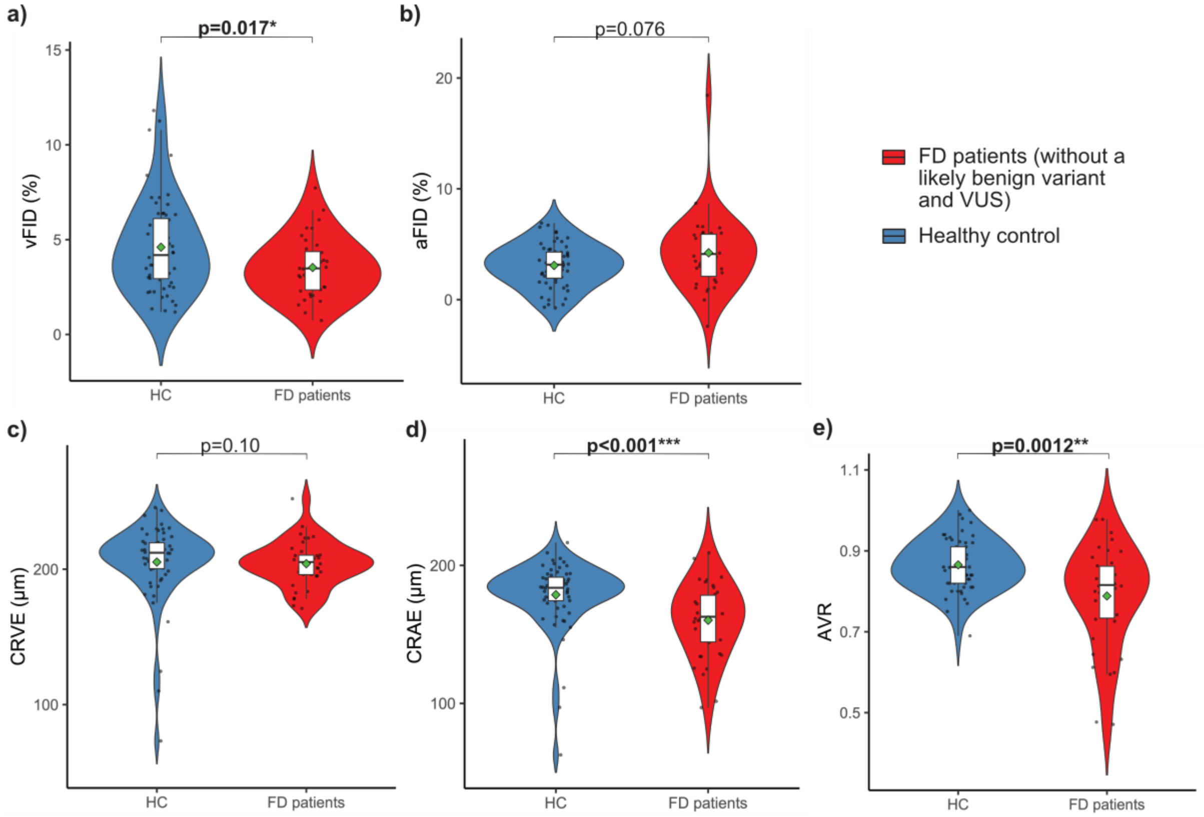

Supplementary Figure 1 Dynamic and static retinal vessel parameters in FD patients without a likely benign or VUS compared to HC Violin plots of DVA parameters, including vFID (a; FD: n=36, HC: n=60) and aFID (b; FD: n=35, HC: n=59), as well as SVA parameters CRVE, CRAE, and AVR (c–e; all FD: n=38 vs. HC: n=60), are shown for age- and Sex-matched FD patients without likely benign variants and VUS (red) and healthy controls (blue). Violinplots display the mean (green square) and median (line). The Wilcoxon rank-sum test was applied for non-parametric distributions, and Student's t-test was used for parametric distributions. Statistical significance is indicated as follows: *p < 0.05; **p < 0.01; ***p < 0.001.

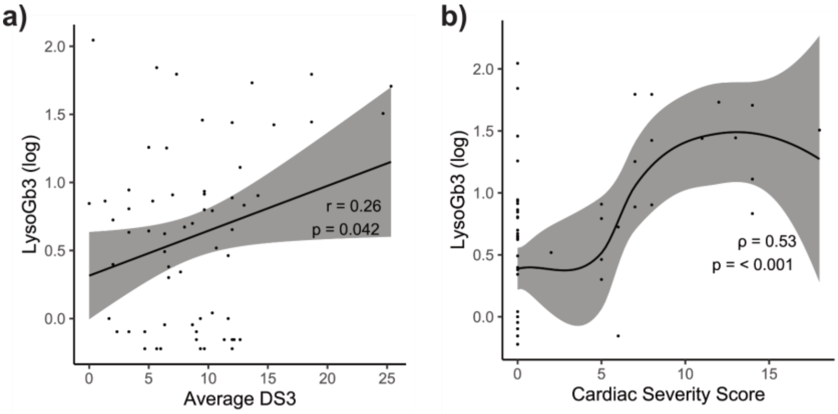

Supplementary Figure 2 Correlation between LysoGb3 with Average DS3 and Cardiac Severity Score

Scatterplots show the Pearson correlations between LysoGb3 (n=63) with Average DS3 (n=63), (a). Spearman correlations are shown for LysoGb3 with the Cardiac Severity Score including the corresponding correlation coefficient (ρ) LOESS curves with 95% confidence intervals are displayed to illustrate the negative, potentially non-linear associations (b).

Dependent variable AVR

Predictors	Univariable		Mutlivariable	
	ß-Coefficient	*P-value*	ß-Coefficient	*P-value*
**Average DS3**	**-0.33**	**0.0098****	-0.24	0.13
**Cardiac Score**	**-0.36**	**0.0045****	**-0.28**	**0.048***
**CNS Score**	**-0.35**	**0.0068****	-0.25	0.092
**Renal Score**	**-0.30**	**0.022***	-0.26	0.060
**CNVD**	**-0.26**	**0.046***	-0.16	0.29
LVH	-0.23	0.074	-0.21	0.13
**HVD**	**-0.45**	**<0.001*****	**-0.44**	**<0.001*****
CHD	-0.099	0.45	-0.061	0.65
MI	-0.065	0.62	-0.061	0.65
HF	0.082	0.54	0.14	0.33
**CKD**	**-0.37**	**0.0035****	**-0.32**	**0.022****

Dependent variable CRVE

Predictors	Univariable		Mutlivariable	
	ß-Coefficient	*P-value*	ß-Coefficient	*P-value*
Average DS3	-0.16	0.22	-0.17	0.26
**Cardiac Score**	**-0.31**	**0.015***	**-0.34**	**0.014***
CNS Score	-0.10	0.47	-0.11	0.48
Renal Score	-0.15	0.27	-0.077	0.57
CNVD	-0.15	0.26	-0.13	0.36
**LVH**	**-0.37**	**0.0041****	**-0.35**	**0.0074****
HVD	0.045	0.73	0.042	0.76
**CHD**	**-0.27**	**0.037***	**-0.26**	**0.046***
MI	-0.14	0.29	-0.11	0.39
HF	-0.11	0.42	-0.041	0.77
CKD	-0.14	0.28	-0.11	0.43

Dependent variable LysoGb3

Predictor	Univariable		Mutlivariable	
	ß-Coefficient	*P-value*	ß-Coefficient	*P-value*
**Average DS3**	**0.26**	**0.042***	**0.31**	**0.040***
**Cardiac Score**	**0.55**	**<0.001****	**0.61**	**<0.001*****
CNS Score	0.16	0.20	0.17	0.26
Renal Score	0.16	0.22	0.17	0.22
**CNVD**	0.15	0.25	0.11	0.44
**LVH**	**0.45**	**<0.001*****	**0.50**	**<0.001*****
**HVD**	**0.34**	**0.0064****	**0.32**	**0.017***
CHD	0.16	0.20	0.15	0.25
MI	0.035	0.78	0.076	0.57
HF	-0.020	0.87	-0.11	0.42
CKD	0.21	0.091	0.20	0.16

Supplementary Table 3 Associations of AVR, CRVE and LysoGb3 with Fabry disease severity scores and cardiac symptom burden Linear regressions with standardized β-coefficients and p-values are shown for CRVE, AVR (both n=60) and LysoGb3 (n=63) in relation to FD disease severity and cardiac involvement. A multivariable model was fitted to adjust for potential confounders, including arterial hypertension, hypercholesterolemia, nicotine abuse, and BMI. The statistical significance is indicated using the following notation: $p < 0.05$ (*), $p < 0.01$ (**), $p < 0.001$ (***).

Clinical characteristics	Likely benigne (n=14)	VUS (n=8)	Likely pathogenic (n=7)	Pathogenic (n=33)	*P value*
**Age**					
mean (SD), years	49.9 (±18.4)	48.4 (±18.0)	47.4 (±10.4)	47.5 (±16.5)	0.65
**Sex**					
female	9 (64.3%)	5 (62.5%)	4 (57.1%)	25 (75.8%)	0.43
**BMI**					
Median (IQR), kg/m^2	25.7 (21.1-30.4)	26.1 (22.8-28.0)	23.0 (20.0-23.5)	24.3 (21.8-27.3)	0.22
**Hypercholesterolemia**	10 (71.4%)	6 (75.0%)	2 (28.6%)	15 (45.5%)	0.15
**Arterial hypertension**	5 (35.7%)	4 (50.0%)	2 (28.6%)	9 (27.2%)	0.65
**Nicotine abuse**	5 (35.7%)	3 (37.5%)	1 (14.3%)	7 (21.2%)	0.77
Fabry-related complications					
**LVH**	3 (21.4%)	4 (50.0%)	5 (71.4%)	17 (51.5%)	0.13
**CHD**	0 (0.0%)	1 (12.5%)	1 (14.3%)	2 (6.1%)	0.36
**HVD**	0 (0.0%)	0 (0.0%)	1 (14.3%)	1 (3.0%)	0.34
**MI**	0 (0.0%)	1 (12.5%)	0 (0.0%)	2 (6.1%)	0.66
**Cardiac arrhythmia**	3 (21.4%)	1 (12.5%)	1 (14.3%)	9 (27.3%)	0.88
**CNVD**	4 (28.6%)	4 (50.0%)	0 (0.0%)	9 (27.3%)	0.40
**CKD**	1 (7.1%)	0 (0.0%)	1 (14.3%)	2 (6.1%)	0.76
**Average DS3**					
Median(IQR)	9.5 (8.7 - 11.6)	8.2 (6.2-11.9)	9.7 (6.5 - 11.2)	6.7 (3.3 - 10.7)	0.55
Laboratory values					
**Leukocytes**					
G/L, IQR	5.9 (5.0 - 6.7)	6.7 (5.2 - 8.0)	5.9 (5.2 - 7.3)	6.6 (5.7 - 7.6)	0.42
**hsCRP**					
mg/L ,Median (IQR)	0.06 (0.02-0.09)	0.13 (0.01-0.14)	0.070 (0.055 – 0.15)	0.13 (0.09-0.24)	**0.038***
**hsTnT**					

ng/L ,Median (IQR)	0.004 (0.001-0.006)	0.015 (0.005-0.028)	0.028 (0.019-0.042)	0.020 (0.008-0.044)	**0.013***
**CK**					
U/L,Median (IQR)	97 (73- 117)	83 (74 -97)	116 (83- 119)	87 (71 - 125)	0.70
**NT-proBNP**					
pg/mL, Mean (SD)	84.1 (±67.5)	392.2 (±793.4)	360.0 (±473.3)	545.4 (±1191.1)	0.63
**LDH**					
U/L, Mean (SD)	192.4 (±37.1)	214.2 (±48.6)	243.4 (±66.1)	223.2 (±64.2)	0.23

Supplementary Table 4 Patients´ characteristics for GLA-gene variant categories P-values are shown for statistical tests comparing the four different GLA-gene variant categories: likely benign (n=14), VUS (n=8), likely pathogenic (n=7), and pathogenic (n=33). ANOVA was used for parametric variables, the Kruskal-Wallis H test for nonparametric variables, the χ^2 test for categorical variables, and Fisher's exact test for proportional variables. BMI, body mass index; hypercholesterolemia, defined as cholesterol > 200 mg/dl; LVH, left ventricular hypertrophy (IVS > 13 mm); CHD, coronary heart disease; HVD, heart valve disease; MI, myocardial infarction; HF, heart failure; CNVD, central nervous vascular disease; CKD, chronic kidney disease (GFR < 60 ml/min); hsCRP, high-sensitivity C-reactive protein (n=56); hsTnT, high-sensitivity troponin T (n=38); CK, creatine kinase (n=63); NT-proBNP, N-terminal prohormone of brain natriuretic peptide (n=38); LDH, lactate dehydrogenase (n=62). The statistical significance of each variant is indicated using the following notation: p < 0.05 (*), p < 0.01 (**), p< 0.001 (***).

Dependent variable LysoGb3$_{log}$

	Univariate			Multivariable		
Predictors	*Estimates*	*CI*	*p*	*Estimates*	*CI*	*p*
Likely pathogenic	1.4	0.9 - 1.8	**<0.001***	1.3	0.91 - 1.7	**<0.001***
Pathogenic	0.9	0.6 – 1.2	**<0.001***	1.0	0.7 – 1.3	**<0.001***
VUS	0.7	0.2 – 1.1	**0.004**	0.6	0.3– 1.0	**0.002**
Age				0.00	-0.01 - 0.01	0.80
Sex [male]				0.51	0.3 - 0.8	**<0.001***

Dependent variable CRAE

	Univariate			Multivariable		
Predictors	*Estimates*	*CI*	*p*	*Estimates*	*CI*	*p*
Likely pathogenic	-28.8	-50.6 – -7.0	**0.011***	-27.9	-46.6 – -9.2	**0.004****
Pathogenic	-14.6	-29.9 – 0.6	0.060	-18.1	-31.4 – -4.9	**0.008****
VUS	-14.3	-35.2 – 6.6	0.18	-14.4	-32.4 – 3.6	0.12
Age				-0.3	-0.65 – 0.00	0.051
Sex[male]				-23.1	-35.5 – -11.4	**<0.001*****

Dependent variable IVS

	Univariate			Multivariable		
Predictors	*Estimates*	*CI*	*p*	*Estimates*	*CI*	*p*
Likely pathogenic	2.4	-1.1 – 6.0	0.17	2.6	0.03 – 5.1	**0.047***
Pathogenic	2.6	0.16 – 5.1	**0.037***	3.3	1.6 – 5.1	**<0.001*****
VUS	2.9	-0.68 – 6.4	0.11	3.4	0.85 – 5.9	**0.010***
Age				0.11	0.06 – 0.16	**<0.001*****
Sex [male]				3.0	1.5 – 4.5	**<0.001*****

Supplementary Table 5 Associations of LysoGb3, CRAE and IVS with variant classification The linear regression model displays estimates, confidence intervals (CI), and p-values for the dependent variables LysoGb3 (n=63), CRAE (n=60), and IVS (n=60), with the classified GLA-gene sequence variants (n=62) as predictors. The multivariable linear model was adjusted for age and sex. The statistical significance of each variant is indicated using the following notation: p < 0.05 (*), p < 0.01 (**), p< 0.001 (***)

IVS as the dependent value

Predictor	Estimate	Std. Error	t Value	P Value
**c.103G>A**	**3.61**	**1.18**	**3.05**	**0.0051****

IVS as the dependent value

Predictor	Estimate	Std. Error	t Value	P Value
c.1064A>T	1.31	2.28	0.57	0.57
c.1090_1103del14bp	3.81	2.98	1.28	0.21
c.1132T>C	2.54	1.98	1.29	0.21
**c.1163_1165del**	**9.67**	**2.86**	**3.38**	**0.0022****
c.1196G>A	4.52	3.06	1.47	0.15
c.125T>C	2.35	2.82	0.84	0.41
c.194+1G>A	3.11	1.99	1.57	0.13
c.241T>G	1.89	2.85	0.66	0.51
**c.281G>T**	**3.75**	**1.77**	**2.12**	**0.043***
c.335G>A	0.82	1.47	0.56	0.58
c.352C>T	-3.64	2.87	-1.27	0.21
c.376A>G	0.80	2.02	0.40	0.70
c.427G>A	1.14	2.76	0.41	0.68
**c.547G>A**	**7.55**	**2.96**	**2.55**	**0.017***
**c.560delT**	**5.18**	**1.76**	**2.95**	**0.0065****
c.568del	5.49	2.88	1.91	0.067
**c.644A>G**	**8.76**	**2.55**	**3.45**	**0.0019****
c.658C>T	4.55	2.80	1.62	0.12
c.671A>G	-2.11	2.68	-0.79	0.44
c.703T>C	0.81	2.84	0.28	0.78
c.783insG	-0.82	2.84	-0.29	0.78
**c.902G>A**	**5.27**	**2.24**	**2.35**	**0.026***
del Exon2 (het.)	4.40	2.90	1.52	0.14

Supplementary Table 6 Associations of IVS with specific GLA-gene sequence variant Multivariable linear regression model showing the relationship between specific GLA-gene sequence variants and IVS thickness, corrected for age and sex and CV risk factors. The refference GLA-gene sequence variant was c.937G>T. The table presents the estimates and p-values for each variant's effect on IVS. Seven gene variants with a potential cardiac phenotype, with the highest positive estimates and significant p-values, are highlighted in red. The statistical significance of each variant is indicated using the following notation: $p < 0.05$ (*), $p < 0.01$ (**)

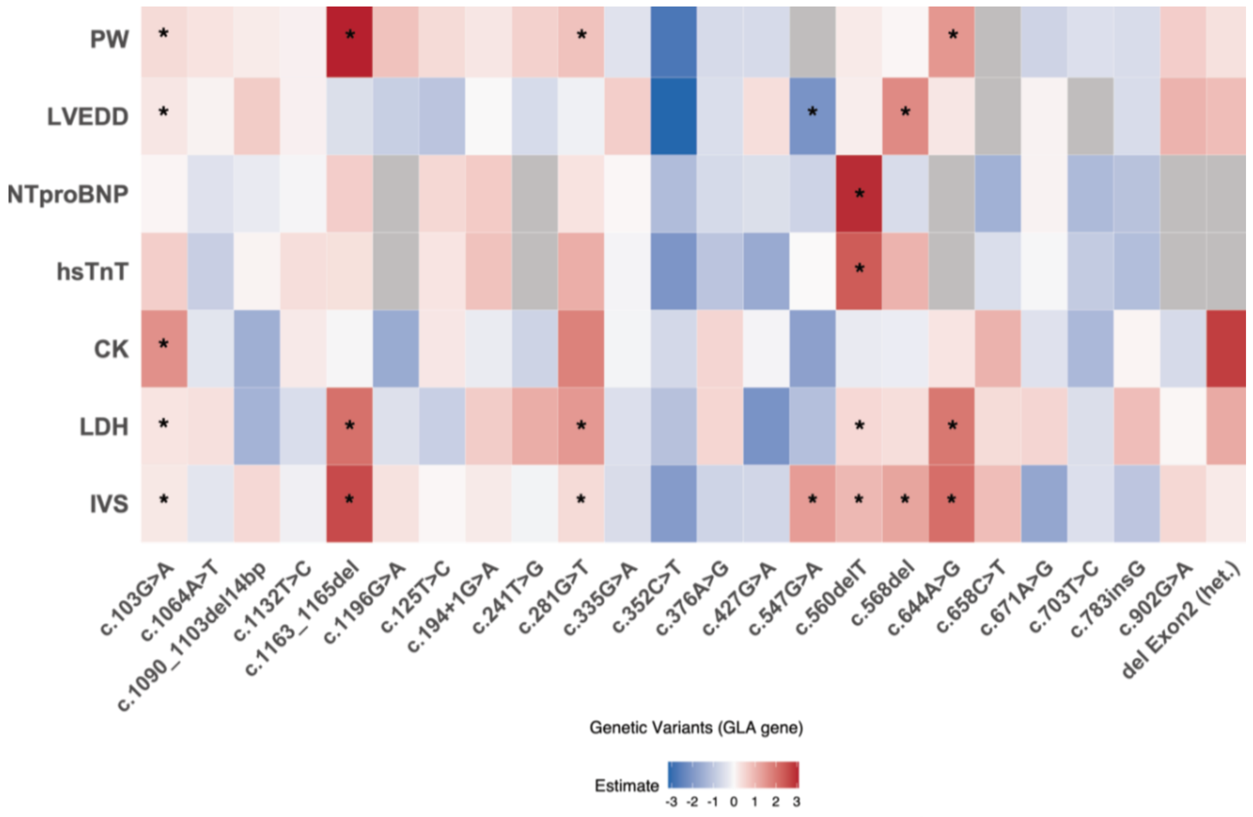

Supplementary Figure 3 Heatmap of scaled estimates across GLA variants for cardiac outcome variables

This heatmap visualizes the scaled estimates of various cardiac outcome variables (y-axis) across GLA variants (x-axis, n=62). The color gradient represents the magnitude of the effect, with red indicating positive scaled estimates and blue indicating negative ones. Neutral estimates are shown in white; grey values indicate missing values. The outcome variables included IVS (n=60), LDH (n=62), CK (n=63), hsTnT (n=38), NT-proBNP (n=38), LVEDD (n=59) and PW (n=58). Statistically significant associations between GLA variants and IVS after correction for age, gender, and CV risk factors in a multivariable linear model are marked with asterisks in the first row. Additionally, significant associations between the potential seven cardiac variants and outcome variables after correction for age, gender, and CV risk factors in a multivariable linear model are marked with asterisks. The color bar shows the range of scaled estimates (ranging from negative=blue to positive=red), with deeper colors corresponding to stronger associations.

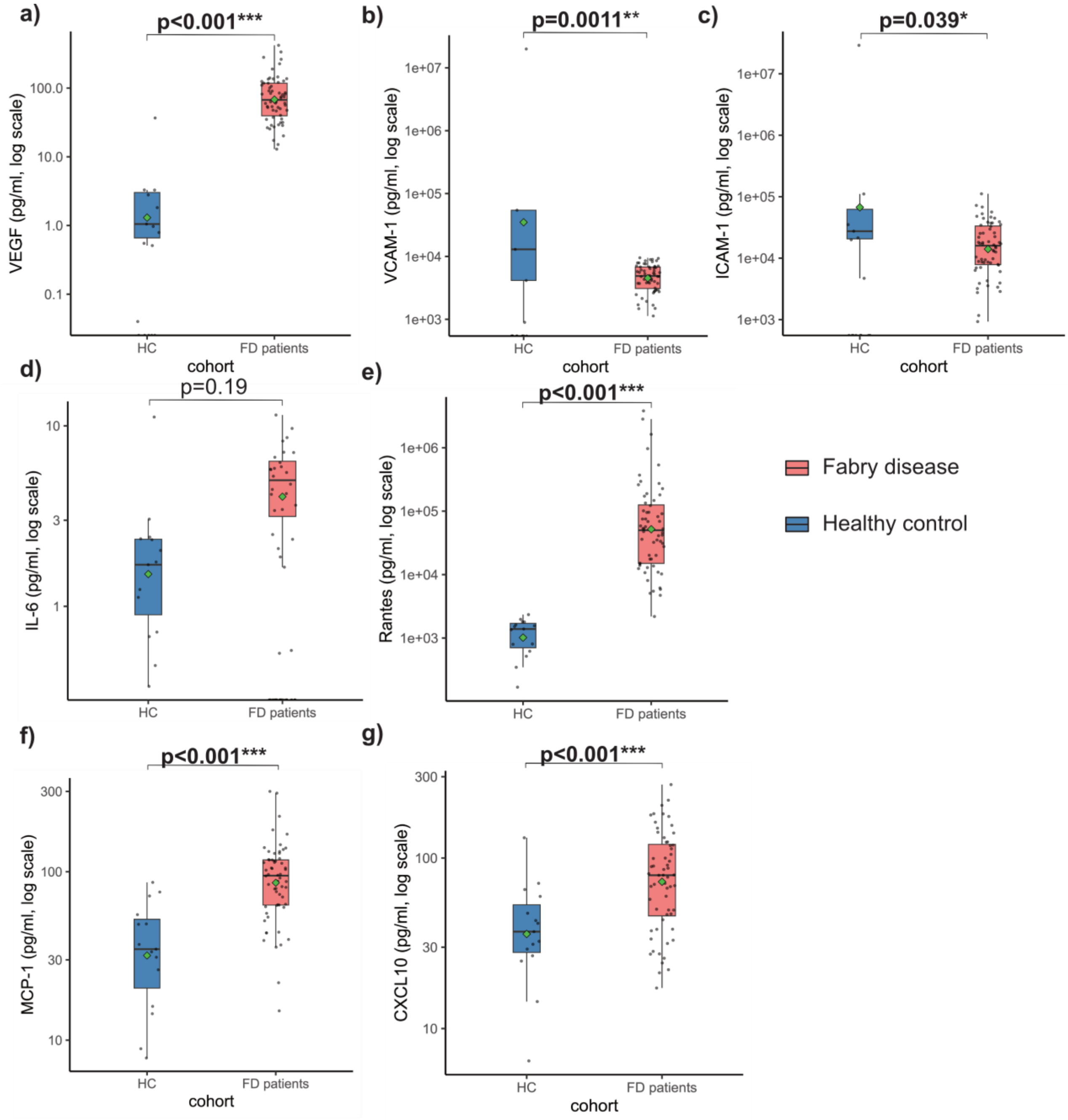

Supplementary Figure 4 Markers of endothelial dysfunction and inflammation in FD patients compared with age and gender-matched HC. Boxplots of laboratory parameters in age- and gender-matched FD patients (red) and healthy controls (light blue). VEGF, RANTES, IL-6, CXCL10, and MCP1 were measured in n=16 HC and n=60 FD patients. ICAM-1 and VCAM-1 were measured in n=15 HC patients and n=60 FD patients. Laboratory values were log-scaled for comparison. Boxplots display the mean (rectangle) and median (line). The Wilcoxon rank-sum test was used for non-parametric distributions, and the Student's t-test was used for parametric distributions. Statistical significance is indicated as follows: $*p < 0.05$; $**p < 0.01$; $***p < 0.001$.

CRAE			
Predictors	*Estimates*	*CI*	*p*
Cardiac Variant x MCP1	-51.28	-104.46 – 1.90	0.058
**Cardiac Variant x CXCL10**	-43.47	-86.57 – -0.38	**0.048***
Cardiac Variant x Rantes	-16.89	-34.79 – 1.01	0.064
Cardiac Variant x IL-6	-1.69	-6.46 – 3.09	0.48
Cardiac Variant x VEGF	-0.23	-51.06 – 50.60	0.99
Cardiac Variant x ICAM-1	-19.58	-49.40 – 10.24	0.19
Cardiac Variant x VCAM-1	-0.00	-0.01 – 0.00	0.084

CRVE			
Predictors	*Estimates*	*CI*	*p*
Cardiac Variant x MCP1	5.62	-34.93 – 46.16	0.78
**Cardiac Variant x CXCL10**	-41.24	-72.8 – -9.7	**0.011***
Cardiac Variant x Rantes	-11.76	-24.9 – 1.44	0.080
Cardiac Variant x IL-6	-0.18	-3.72 – 3.35	0.92
Cardiac Variant x VEGF	-16.62	-52.02 – 18.78	0.35
Cardiac Variant x ICAM-1	-19.70	-41.53 – 2.12	0.076
Cardiac Variant x VCAM-1	-0.00	-0.01 – 0.00	0.18

AVR			
Predictors	*Estimates*	*CI*	*p*
**Cardiac Variant x MCP1**	-0.26	-0.51 – -0.01	**0.042***
Cardiac Variant x CXCL10	-0.06	-0.27 – 0.15	0.56
Cardiac Variant x Rantes	-0.03	-0.12 – 0.05	0.45
Cardiac Variant x IL-6	-0.01	-0.03 – 0.01	0.47
Cardiac Variant x VEGF	0.05	-0.18 – 0.29	0.66
Cardiac Variant x ICAM-1	-0.01	-0.16 – 0.13	0.85
Cardiac Variant x VCAM-1	-0.00	-0.00 – 0.00	0.39

$LysoGb3_{log}$			
Predictors	*Estimates*	*CI*	*p*
**Cardiac Variant x MCP1**	1.48	0.16 – 2.59	**0.010***
Cardiac Variant x CXCL10	0.00	-0.01 – 0.01	0.79
Cardiac Variant x Rantes	0.31	-0.13 – 0.75	0.17
Cardiac Variant x IL-6	-0.05	-0.16 – 0.06	0.40
Cardiac Variant x VEGF	0.07	-0.96 – 1.10	0.89
Cardiac Variant x ICAM-1	0.32	-0.32 – 0.95	0.32
Cardiac Variant x VCAM-1	0.00	-0.00 – 0.00	0.23

Supplementary Table 7 Interaction models between static retinal parameters, laboratory values and cardiac variants in Fabry patients The models represent multivariable linear regression analyses with AVR, CRAE, CRVE (n=60) and LysoGb3 (n=63) as the dependent variables. The interaction terms between the potential cardiac variant (n=20) and laboratory variables (MCP1, CXCL10, RANTES, IL-6, VEGF, ICAM-1, and VCAM-1, all n=60) in Fabry patients serve as the predictor variables. All laboratory values except VCAM-1 were log-transformed prior to fitting the models. CI indicates Confidence Interval.

ID	GLA-Gene NM_000169.3	Sequence variance	Classification	Variant classification
1	c.376A>G	p.S126G	2	likely benign
2	c.376A>G	p.S126G	2	likely benign
3	c.937G>T	p.Asp313Tyr	2	likely benign
4	c.125T>C	p.Met42Thr	5	pathogenic
5	c.560delT	p.Met187fs	5	pathogenic
6	c.937G>T	p.Asp313Tyr	2	likely benign
7	c.937G>T	p.Asp313Tyr	2	likely benign
8	c.103G>A	p.Gly35Arg	5	pathogenic
9	c.103G>A	p.Gly35Arg	5	pathogenic
10	c.902G>A	p.Asp301Gln	3	VUS
11	c.783insG	p.Trp262fs	5	pathogenic
12	c.427G>A	p.Ala143Thr	3	VUS
13	c.103G>A	p.Gly35Arg	5	pathogenic
14	c.902G>A	p.Asp301Gln	3	VUS
15	c.1132T>C	p.Cys378ARG	4	likely pathogenic
16	c.352C>T	p.Arg118Cys	3	VUS
17	c.352C>T	p.Arg118Cys	3	VUS
18	c.560delT	p.Phe169fs	5	pathogenic
19	c.937G>T	p.Asp313Tyr	2	likely benign
20	c.1196G>A	p.W399	5	pathogenic
21	c.1163_1165del	p.Leu388del	3	VUS
22	c.658C>T	p.Arg220	5	pathogenic
23	c.281G>T	p.Cys94Phe	4	likely pathogenic
24	c.194+1G>A	Splice- Variance	5	pathogenic
25	c.103G>A	p.Gly35Arg	5	pathogenic
26	c.194+1G>A	Splice- Variance	5	pathogenic
27	c.194+1G>A	Splice- Variance	5	pathogenic
28	c.937G>T	p.Asp313Tyr	2	likely benign

29	c.1132T>C	p.Cys378ARG	4	likely pathogenic
30	c.671A>G	p.Asn224Ser	5	pathogenic
31	del Exon2 (het.)		5	pathogenic
32	c.281G>T	p.Cys94Phe	4	likely pathogenic
33	c.281G>T	p.Cys94Phe	4	likely pathogenic
34	c.103G>A	p.Gly35Arg	5	pathogenic
35	c.937G>T	p.Asp313Tyr	2	likely benign
36	c.703T>C	p.Ser235Pro	4	likely pathogenic
37	c.335G>A	p.Arg112His	5	pathogenic
38	c.335G>A	p.Arg112His	5	pathogenic
39	c.937G>T	p.Asp313Tyr	2	likely benign
40	c.937G>T	p.Aps313Tyr	2	likely benign
41	c.937G>T	p.Asp313Tyr	2	likely benign
42	c.937G>T	p.Asp313Tyr	2	likely benign
43	c.335G>A	p.Arg112His	5	pathogenic
44	c.335G>A	p.Arg112His	5	pathogenic
45	Missing			
46	c.568del	p.Ala190Profs*2 (p.Ala190fs)	5	pathogenic
47	c.335G>A	p.Arg112His	5	pathogenic
48	c.241T>G	p.Trp81Gly	4	likely pathogenic
49	c.937G>T	p.Asp313Tyr	2	likely benign
50	c.1064A>T	p.Asn355Ile	3	VUS
51	c.1064A>T	p.Asn355Ile	3	VUS
52	c.335G>A	p.Arg112His	5	pathogenic
53	c.103G>A	p.Gly35Arg	5	pathogenic
54	c.103G>A	p.Gly35Arg	5	pathogenic
55	c.568del	p.Ala190Profs*2	5	pathogenic
56	c.547G>A	IVS3+1G>A	5	pathogenic
57	c.1090_1103del14bp	p.Tyr365Cysfs*5 (p.Tyr365fs)	5	pathogenic
58	c.644A>G	p.Asn215Ser	5	pathogenic
59	c.644A>G	p.Asn215Ser	5	pathogenic
60	c.560delT	p.Phe169fs	5	pathogenic
61	c.937G>T	p.Asp313Tyr	2	likely benign
62	c.103G>A	p.Gly35Arg	5	pathogenic
63	c.103G>A	p.Gly35Arg	5	pathogenic

Supplementary Table 8 Classification of GLA-gene variants according to the American College of Medical Genetics and Genomics (ACMG) guideline Table presents GLA gene variants, the corresponding sequence variants, and their classification based on ACMG guidelines in 62 FD patients.
